# T cell immunity to Zika virus targets immunodominant epitopes that show cross-reactivity with other Flaviviruses

**DOI:** 10.1038/s41598-017-18781-1

**Published:** 2018-01-12

**Authors:** C. J. Reynolds, O. M. Suleyman, A. M. Ortega-Prieto, J. K. Skelton, P. Bonnesoeur, A. Blohm, V. Carregaro, J. S. Silva, E. A. James, B. Maillère, M. Dorner, R. J. Boyton, D. M. Altmann

**Affiliations:** 1 Department of Medicine, Imperial College London, London, United Kingdom; 2 CEA-Saclay, Institute Frédéric Joliot, Gif Sur Yvette, France; 3 Department of Biochemistry and Immunology, University of São Paulo, Ribeirão Preto, Brazil; 4 Benaroya Research Institute at Virginia Mason, Seattle, Washington United States of America

## Abstract

Zika virus (ZIKV) Infection has several outcomes from asymptomatic exposure to rash, conjunctivitis, Guillain-Barré syndrome or congenital Zika syndrome. Analysis of ZIKV immunity is confounded by the fact that several related Flaviviruses infect humans, including Dengue virus 1–4, West Nile virus and Yellow Fever virus. HLA class II restricted T cell cross-reactivity between ZIKV and other Flaviviruses infection(s) or vaccination may contribute to protection or to enhanced immunopathology. We mapped immunodominant, HLA class II restricted, CD4 epitopes from ZIKV Envelope (Env), and Non-structural (NS) NS1, NS3 and NS5 antigens in HLA class II transgenic mice. In several cases, ZIKV primed CD4 cells responded to homologous sequences from other viruses, including DENV1–4, WNV or YFV. However, cross-reactive responses could confer immune deviation - the response to the Env DENV4 p1 epitope in HLA-DR1 resulted in IL-17A immunity, often associated with exacerbated immunopathogenesis. This conservation of recognition across Flaviviruses, may encompass protective and/or pathogenic components and poses challenges to characterization of ZIKV protective immunity.

## Introduction

Zika virus (ZIKV) is a Flavivirus, first discovered in 1947 in Uganda and named after the Zika Forest^1–3^. The current outbreak has been associated also with a large increase in birth defects, ‘congenital syndrome associated with Zika virus infection’, notably microcephaly^1,2^.

There is a pressing need to understand innate and adaptive immune mechanisms protecting against the virus. Human immunity to this virus had not been extensively researched until the current outbreak and analysis has posed important challenges. With several Zika vaccine candidates poised to enter trials, there is an imperative to have agreed measurables to serve as correlates of protection^4^. In ZIKV infection (as indeed for most other Flavivirus infections) we lack the datasets of T cell response patterns that might offer us the immune correlates of differential outcome after exposure; this is equally true whether considering outcomes after natural exposure or in vaccine trials. Neutralizing antibodies, CD4 and CD8 immunity might be envisaged as important. However, knowledge of immunity to other related Flavivruses such as West Nile virus (WNV), Japanese encephalitis virus (JEV) and Dengue virus (DENV), suggests that it may be predicted that components of anti-viral antibody, CD4 or CD8 responses could themselves be highly pathogenic^5–8^. The pathogenesis of both WNV and JEV infection encompasses T cell mediated pathology^5–7^. Another confounder to simple analysis of Zika virus (ZIKV) immunity is the issue of virus cross-reactivity. In several of the countries in which ZIKV currently poses a threat, there may be as many as 10 different, related Flavivruses circulating, including DENV serotypes 1–4 and Yellow Fever virus (YFV), as well as YFV vaccination being widespread^9^. This raises important issues. First, that antigenic cross-reactivity has imposed a confounder for design and roll-out of reagents for unequivocal serodiagnostics and, therefore, for seroprevalence reporting^10^. Second, that the impact of anti-viral cross-reactivity has not been taken into account. A simple view would be that a high level of immunity in these populations to some conserved or partially conserved antigen sequences may offer a degree of protection from ZIKV infection. An alternative, non-mutually exclusive hypothesis, is that as with antibody dependent enhancement (ADE) between DENV serotypes, prior immunity to related viruses may potentiate a pathogenic response to subsequent ZIKV exposure or vaccination (or indeed, that ZIKV immunity will alter responses to subsequent challenges with viruses such as DENV or YFV)^4,11^. Such considerations make detailed mapping of interactions between the respective anti-viral repertoires a priority.

Recent studies from mouse models, non-human primates and exposed humans have started to describe features of adaptive immunity to ZIKV^4,11–16^. Furthermore, clues can be extrapolated from previous studies with other Flaviviruses^17^. Attempts to model acute infection in mice have encompassed neonatal or in utero infection of wild-type strains as well as infection of type I interferon receptor knockout strains. These models variably involve central nervous system (CNS) infiltration by CD4 (mainly T helper 1 (Th1)) and CD8 T cells, sometimes with associated neuropathology. In two separate studies, analysis of ZIKV CD8 IFNγ production highlighted an immunodominant epitope from envelope, in the region of amino acids 294–302 of the full length viral sequences^13,14^. Work by Elong Ngono and colleagues showed that murine ZIKV acute infection leads to an increase in activated CD8 T cells^14^. This response was implicated in protection since CD8 ablation exacerbated disease and transfer of primed CD8 T cells decreased viral load. Epitope mapping of CD8 responses by screening recall responses to ZIKV peptides, predicted using the www.iedb.org predictive tools, defined epitopes in Env, prM and NS5 proteins, including the aforementioned Env 294–302 epitope. Acute infection of rhesus macaques showed viral persistence at several sites including CNS (with CNS lymphocyte infiltration), CD4 and CD8 cells activation and a neutralizing antibody response targeted largely to envelope and NS1^18^. A study of ZIKV immunity in ZIKV-exposed humans indicated that while antigenic cross-reactivity between DENV and ZIKV was evident in the antibody repertoire, this was less apparent in the T cell response to Env and NS1^12^.

We set out to put in place an initial dataset of Human leukocyte antigen (HLA) class II restricted responses to ZIKV epitopes that may facilitate analysis of correlates of protection. We used transgenic mouse models that express human class II with a particular focus on HLA-DRB1*0101 and HLA-DRB1*0401 since these alleles are relatively common in ZIKV exposed communities in the Americas. For example, the allele frequencies of HLA-DRB1*0101 and HLA-DRB1*0401 in Sao Paulo, Brazil are respectively, 0.100 and 0.119 (n = 800,809) www.allelefrequencies.net. Furthermore, we looked for evidence that ZIKV-primed CD4 T cells may recognize cross-reactive epitopes from the homologous antigens in other Flaviviruses. Using immunization of HLA class II transgenics with ZIKV antigen, and then acute infection of AG129 mice, we describe a large array of ZIKV CD4 T cell epitopes, the most comprehensive thus far. ZIKV primed T cells can in some cases respond to related epitopes from other Flaviviruses. However, this cross-reactive recognition can result in ‘immune deviation’ of the response to an altered cytokine profile of enhanced Interleukin 17 A (IL-17A). This argues for the potential of prior ZIKV infection or vaccination to skew subsequent immunopathogenesis on exposure to DENV.

## Results

### Mapping immunodominant CD4 T cell ZIKV epitopes in HLA class II transgenic mice

As a first step to the definition of HLA-restricted, CD4 T cell epitopes from ZIKV, we immunized transgenic mice that carried human class II molecules (all on an H2Aβ −/− background), with recombinant ZIKV antigens NS1, NS3, NS5 and Env (Table S1) and determined recall T cell responses to the full sequence as peptide 20mers overlapping by 10 amino acids (Table S2). The decision to focus initial efforts on NS1, NS3, NS5 and Env was based on extrapolation from the ranking of immunogenicity of CD4 T cell antigens in WNV^6^. HLA-DR1 and HLA-DR4 transgenics were immunized with ZIKV NS1 and recall T cell responses to the full sequence of peptide 20mers overlapping by 10 amino acids analysed. One CD4 epitope was identified in HLA-DR1 transgenics (p28) and one in HLA-DR4 (p18). Immunization with NS3 demonstrated one epitope presented by HLA-DR1 (p15) and 5 by HLA-DR4 (p12, 28, 29, 42, 51). Immunization with NS5 showed no HLA-DR1 epitopes and 5 HLA-DR4 epitopes (p22, 23, 26, 28, 29) (Table 1 and Fig. S1). We had thus shown, with respect to HLA-DR1 and DR4 restricted epitopes from NS1, 3 and 5, that we could define 13 epitopes, 2 from NS1, 6 from NS3, and 5 from NS5.

**Table 1:** CD4 T cell epitopes identified from the Zika virus protein antigens NS1, NS3, and NS5 in HLA-DR1, and -DR4, transgenic lines.

HLA transgenic strain	ZIKV antigen, T cell epitopes identified		
	NS1	NS3	NS5
HLA-DR4	p18 [171–190]	p12 [111–130]p28 [271–190]p29 [281–300]p42 [411–430]p51 [501–520]	p22 [211–230]p23 [221–240]p26 [251–270]p28 [271–290]p29 [281–300]
HLA-DR1	p28 [271–290]	p15 [141–160]	—

**Figure 1:**
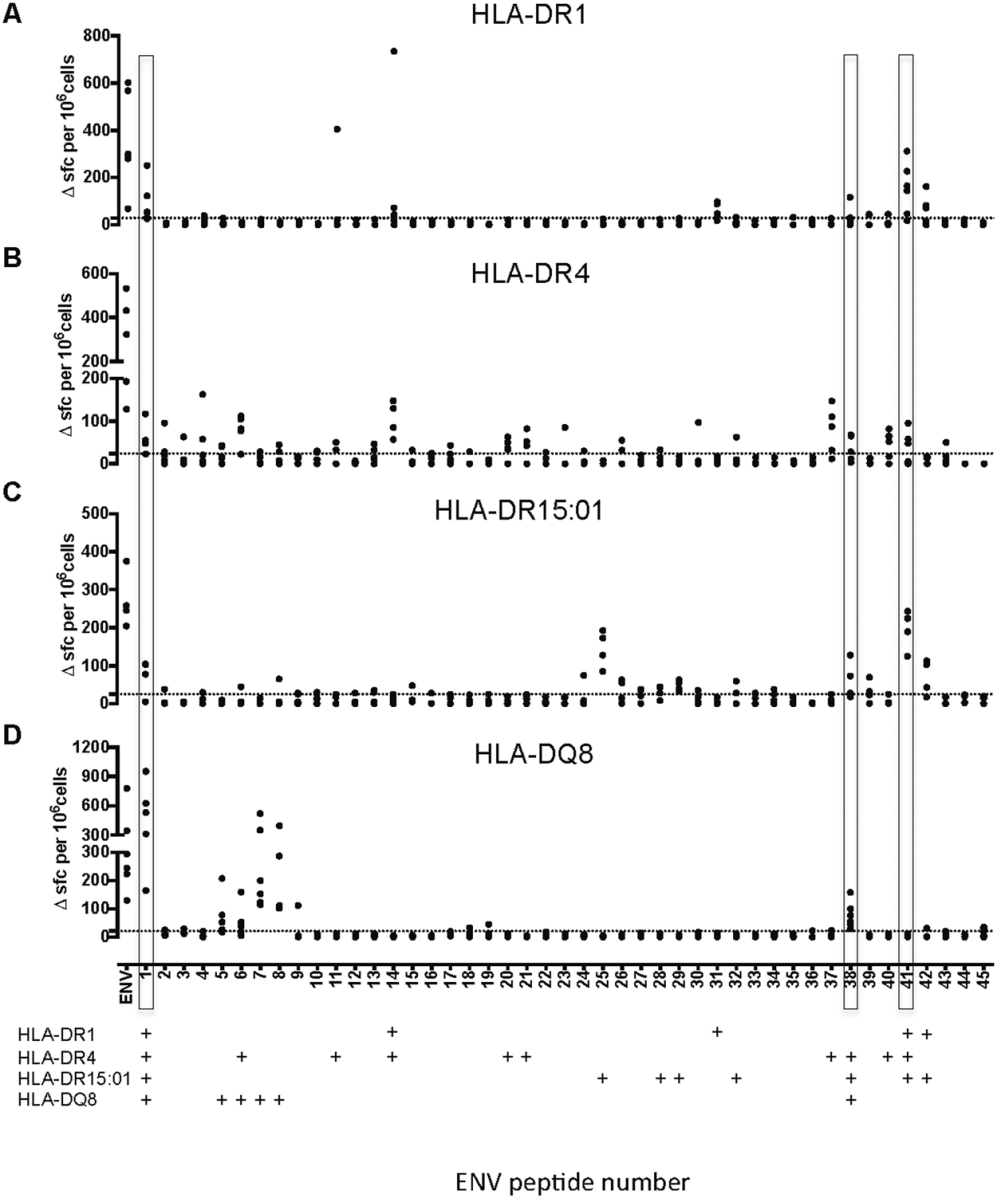
CD4 T–cell epitopes to Zika envelope protein in HLA class II transgenic mice. Mice transgenic for HLA-DR1 (DRB1*0101) n = 6 (**A**); HLA-DR4 (DRB1*0401) n = 5 (**B**); HLA-DR15:01 (DRB1*1501) n = 4 (**C**) and HLA-DQ8 (DQB1*0302) n = 6 (**D**) were primed with 25 μg of recombinant zika envelope protein (Env). 10 days after immunisation, draining lymph node cells (DLNs) were assayed by IFNγ ELISpot for recall responses to the Env protein and to an overlapping panel of 50 Env peptides. Data are plotted as number of spot forming cells (SFC) per 10^6^ cells for individual mice. Responses were considered positive (+) if the response was greater than 2 SD above the mean of the response in the absence of any antigen (shown as horizontal dotted line). Peptides that were defined as positive epitopes across 3 or more HLA class II transgenic mouse lines are highlighted.

T cell epitopes from Env were investigated more broadly, by immunization of mice transgenic for HLA-DR1, DR4, DR1501 or DQ8 (Fig. 1). This enabled us to define a further 29 peptide/HLA combinations. Env was the most CD4 epitope-rich of the antigens. Interestingly, some epitopes were presented by more than one class II heterodimer: for example, Env p1 and p41 were presented by HLA-DR1, DR4, and DR1501, while p1 and p38 were presented by HLA-DR4, HLA-DR1501 and HLA-DQ8. The definition of epitopes that can be presented through both HLA-DR and HLA-DQ peptide binding grooves is interesting in light of the relatively distinct structures and binding pockets of these heterodimers. However, these peptides may be presented in different registers by these structurally distinct heterodimers. Env p1 was here found to be presented by HLA-DR1, DR4, DR1501 and DQ8, as well as overlapping the sequence described by others as a mouse class I binder for stimulation of CD8 T cells. That is, peptide 1–20 of Env overlies the CD8 epitope previously termed 294–302 in the full viral sequence^13,14^. As might be predicted from this, the IFNγ T cell signal in our assays with p1 was found to derive both from CD4 cells and from (endogenous class I restricted) CD8 cells (Fig. S2), despite the fact that a 20mer peptide would not be considered well suited to exogenous loading via the class I pathway. However, it is possible that some of the peptide taken up by the cell exogenously is able to be re-processed and access the class I presentation pathway.

To summarize the ZIKV T cell epitopes characterized by our approach, we identified 42 different peptide-HLA combinations. Of the antigens that we epitope mapped, Env was by far the most CD4 epitope-rich: taking the example of HLA-DR4 presentation, Env carries 2.03 CD4 epitopes for every 10 kDa protein sequence, followed by 0.73 for NS3, 0.49 for NS5 and 0.25 for NS1 (Table 2).

**Table 2:** Epitope density of ZIKV proteins.

HLA transgenic strain	ZIKV antigen, Epitope richness (no. of epitopes [epitopes per 10 kDa])			
	Env	NS1	NS3	NS5
HLA-DR4	10 [2.03]	1 [0.25]	5 [0.73]	5 [0.49]
HLA-DR1	5 [1.02]	1 [0.25]	1 [0.14]	0 [0]

We further investigated the extent to which some of the CD4 epitopes we had defined might be broadly recognized by human T cells by conducting peptide binding assays to a panel of seven different HLA-class II heterodimers. The peptides tested had been identified in the HLA-DR1 transgenic line. Env p1 was also positive in HLA–DR4, DR1501, and DQ8 transgenics; Env p14 was also positive in HLA–DR4 transgenics; Env p41 was also positive in HLA-DR4 and −1501 transgenics; and Env p42 was also positive in HLA-DR1501 (Fig. 1). Each of the 5 peptides showed moderate to high relative binding affinity to at least one of the heterodimers tested (Table 3). P14 and p31 showed moderate to strong relative binding affinity to almost all the HLA-class II molecules tested. Such peptides will be strong candidates for screening of ZIKV vaccinated cohorts, for example using ELISpot or class II tetramer analysis.

**Table 3:** ZIKV Env peptide relative binding affinity to HLA-DR molecules.

Env peptide	DR1	DR3	DR4	DR7	DR11	DR15	DRB5
p1 [1–20]	638	**8**	118	86	>2 248	175	474
p14 [131–150]	**1**	250	**0.2**	**7**	**0.4**	**0.2**	**1**
p31 [301–320]	**3**	>1 129	**11**	**1**	50	53	**2**
p41 [401–420]	900	>1 129	114	>359	**4**	75	65
p42 [411–430]	319	**19**	>477	119	**5**	125	54

Data were expressed as relative affinity: ratio of the half maximal inhibitory concentration of the peptide to the half maximal inhibitory concentration of the reference peptide. The peptide concentration preventing 50% binding of labeled peptide (IC50) was evaluated and data expressed as relative affinity: the ratio of the IC50 of test peptide to the IC50 of reference peptide. Mean ± SEM was calculated from two to three independent experiments. Relative affinities of 10 or less were considered high binders, and relative affinities of 10–100 were moderate binders.

### Cross-reactive T cell epitope recognition between ZIKV and other Flaviviruses

The T cell epitope sequences identified above were submitted to a BLAST search to identify other viruses containing partially homologous sequences. To analyse potential T cell cross-reactivity between the ZIKV epitopes and epitopes from other Flaviviruses, we aligned the sequences of ZIKV Env p1, p6, p7, p8, p14, p25, p29, p31, p32, p38, p41 and p42 against those of DENV1–4, WNV and YFV. The aligned virus sequences contained partially homologous sequences with an amino acid match at 3 to 17 residues out of 20. We synthesized the variant Env epitopes from the sequences of WNV, YFV and DENV1–4, for p1, p6, p7, p8, p14, p25, p29, p31, p,32, p38, p41 and p42 (Table 4).

**Table 4:** Aligned the sequences of ZIKV Env p1, p6, p7, p8, p14, p29, p31, p32, p38, p41 and p42 against those of DENV 1–4, WNV and YFV.

Peptide name	Peptide Sequence	Sequence Identity
**Peptide 1 variants**		
ZIKV P1	IRCIGVSNRDFVEGMSGGTW	
WNV P1	FN**C**L**G**M**SNRDF**L**EG**V**SG**A**TW**	13/20
YFV P1	AH**CIG**ITD**RDF**I**EG**VH**GGTW**	12/20
DENV1 P1	M**RC**V**G**IG**NRDFVEG**L**SG**A**TW**	14/20
DENV2 P1	M**RCIG**I**SNRDFVEG**V**SGG**S**W**	16/20
DENV3 P1	M**RC**V**GV**G**NRDFVEG**L**SG**A**TW**	15/20
DENV4 P1	M**RC**V**GV**G**NRDFVEG**V**SGG**A**W**	15/20
**Peptide 6 variants**		
ZIKV P6	SNMAEVRSYCYEASISDMAS	
WNV P6	A**N**L**AEVRSYCY**L**A**TV**SD**LST	12/20
YFV P6	DGP**AE**A**R**KV**CY**S**A**VLTNVKI	6/20
DENV1 P6	T**N**P**A**VL**R**KL**C**I**EA**K**IS**NTTT	8/20
DENV2 P6	KQP**A**TL**R**K**YC**I**EA**KLTNTTT	6/20
DENV3 P6	TQL**A**TL**R**KL**C**I**E**GK**I**TNITT	5/20
DENV4 P6	KEV**A**LL**R**T**YC**I**EA**L**IS**NITT	8/20
**Peptide 7 variants**		
ZIKV P7	YEASISDMASDSRCPTQGEA	
WNV P7	**Y**L**A**TV**SD**LSTKAA**CPT**M**GEA**	10/20
YFV P7	**Y**S**A**VLTNVKINDK**CP**ST**GEA**	7/20
DENV1 P7	I**EA**K**IS**NTTT**DSRCPTQGEA**	14/20
DENV2 P7	I**EA**KLTNTTTE**SRCPTQGE**P	10/20
DENV3 P7	I**E**GK**I**TNITT**DSRCPTQGEA**	12/20
DENV4 P7	I**EA**L**IS**NITTAT**RCPTQGE**P	11/20
**Peptide 8 variants**		
ZIKV P8	DSRCPTQGEAYLDKQSDTQY	
WNV P8	KAA**CPT**M**GEA**HN**DK**RA**D**PAF	9/20
YFV P8	NDK**CP**ST**GEA**H**L**EEENEGDN	6/20
DENV1 P8	**DSRCPTQGEA**T**L**VEEQ**D**ANF	12/20
DENV2 P8	E**SRCPTQGE**PS**L**NEEQ**D**KRF	10/20
DENV3 P8	**DSRCPTQGEA**V**L**PEEQ**D**QN**Y**	13/20
DENV4 P8	AT**RCPTQGE**P**YL**KEEQ**D**Q**QY**	12/20
**Peptide 14 variants**		
ZIKV P14	QPENLEYRIMLSVHGSQHSG	
WNV P14	LK**EN**IK**Y**EVAIF**VHG**PTTVE	6/20
YFV P14	DQTKIQ**Y**V**I**RAQL**H**VGAKQE	3/20
DENV1 P14	**Q**Y**ENL**K**Y**SVIVT**VH**TGDQHQ	7/20
DENV2 P14	**QPENLEY**T**I**VITP**H**SGEEHA	9/20
DENV3 P14	**Q**Y**ENL**K**Y**TVIIT**VH**TGDQHQ	7/20
DENV4 P14	**Q**I**ENLEY**TVVVT**VH**NGDTHA	8/20
**Peptide 25 variants**		
ZIKV P25	ALVEFKDAHAKRQTVVVLGS	
WNV P25	T**L**M**EF**EEP**HA**TK**Q**S**V**IA**LGS**	10/20
YFV P25	H**LVEF**EPP**HA**ATIK**V**LA**LG**N	9/20
DENV1 P25	L**LV**T**FK**T**AHAK**K**Q**E**VVVLGS**	15/20
DENV2 P25	T**LV**T**FK**NP**HAK**K**Q**D**VVVLGS**	14/20
DENV3 P25	L**LV**T**FK**N**AHAK**K**Q**E**VVVLGS**	15/20
DENV4 P25	RM**V**T**FK**VP**HAKRQ**D**V**T**VLGS**	13/20
**Peptide 29 variants**		
ZIKV P29	KGRLSSGHLKCRLKMDKLRL	
WNV P29	TVK**L**T**SGHLKCR**V**KM**E**KL**Q**L**	13/20
YFV P29	LYK**L**HG**GH**VA**CR**V**K**LSA**L**T**L**	8/20
DENV1 P29	TTTIFA**GHLKCRLKMDKL**T**L**	13/20
DENV2 P29	GNL**L**FT**GHLKCRL**R**MDKL**Q**L**	13/20
DENV3 P29	GTSIFA**GHLKCRLKMDKL**E**L**	13/20
DENV4 P29	GNHMFA**GHLK**CKVR**M**E**KL**RI	7/20
**Peptide 31 variants**		
ZIKV P31	KGVSYSLCTAAFTFTKIPAE	
WNV P31	**KG**TT**Y**GV**C**SK**AF**K**F**LGT**PA**D	9/20
YFV P31	**KG**T**SY**KM**CT**DKMS**F**V**K**N**P**TD	9/20
DENV1 P31	**KG**M**SY**VM**CT**GS**F**KLE**K**EV**AE**	10/20
DENV2 P31	**KG**M**SYS**M**CT**GK**F**KVV**K**EI**AE**	11/20
DENV3 P31	**KG**M**SY**AM**CT**NT**F**VLK**K**EVS**E**	9/20
DENV4 P31	**KG**M**SY**TM**C**SGK**F**SID**K**EM**AE**	9/20
**Peptide 32 variants**		
ZIKV P32	AFTFTKIPAETLHGTVTVEV	
WNV P32	**AF**K**F**LGT**PA**D**T**G**HGTV**VL**E**L	11/20
YFV P32	KMS**F**V**K**N**P**TD**T**G**HGT**AVMQ**V**	8/20
DENV1 P32	S**F**KLE**K**EV**AET**Q**HGTV**L**V**Q**V**	11/20
DENV2 P32	K**F**KVV**K**EI**AET**Q**HGT**IVIR**V**	9/20
DENV3 P32	T**F**VLK**K**EVS**ET**Q**HGT**ILIK**V**	8/20
DENV4 P32	K**F**SID**K**EM**AET**Q**HGT**TV**V**K**V**	10/20
**Peptide 38 variants**		
ZIKV P38	NSKMMLELDPPFGDSYIVIG	
WNV P38	**N**A**K**VLI**EL**E**PPFGDSYIV**V**G**	14/20
YFV P38	DDEVLI**E**VN**PPFGDSYI**I**VG**	11/20
DENV1 P38	EKPVNI**E**TE**PPFG**E**SYIV**V**G**	10/20
DENV2 P38	D**S**PVNI**E**AE**PPFGDSYI**IV**G**	11/20
DENV3 P38	DEPVNI**E**AE**PPFG**E**S**N**IVIG**	10/20
DENV4 P38	**NS**VTNI**EL**E**PPFGDSYIVIG**	15/20
**Peptide 41 variants**		
ZIKV P41	HRSGSTIGKAFEATVRGAKR	
WNV P41	**H**K**SGS**S**IGKAF**TT**T**LK**GA**Q**R**	13/20
YFV P41	**H**KE**GS**S**IGK**L**F**TQ**T**MK**GA**E**R**	11/20
DENV1 P41	FKK**GS**S**IGK**M**FEAT**A**RGA**R**R**	13/20
DENV2 P41	FKK**GS**S**IG**QM**FE**T**T**M**RGAKR**	12/20
DENV3 P41	YKK**GS**S**IGK**M**FEAT**A**RGA**R**R**	13/20
DENV4 P41	F**R**K**GS**S**IGK**M**FE**S**T**Y**RGAKR**	14/20
**Peptide 42 variants**		
ZIKV P42	FEATVRGAKRMAVLGDTAWD	
WNV P42	**F**TT**T**LK**GA**Q**R**L**A**A**LGDTAWD**	13/20
YFV P42	**F**TQ**T**MK**GA**E**R**L**AV**M**GD**A**AWD**	12/20
DENV1 P42	**FEAT**A**RGA**R**RMA**I**LGDTAWD**	17/20
DENV2 P42	**FE**T**T**M**RGAKRMA**I**LGDTAWD**	17/20
DENV3 P42	**FEAT**A**RGA**R**RMA**I**LGDTAWD**	17/20
DENV4 P42	**FE**S**T**Y**RGAKRMA**I**LG**E**TAWD**	16/20

**Figure 2:**
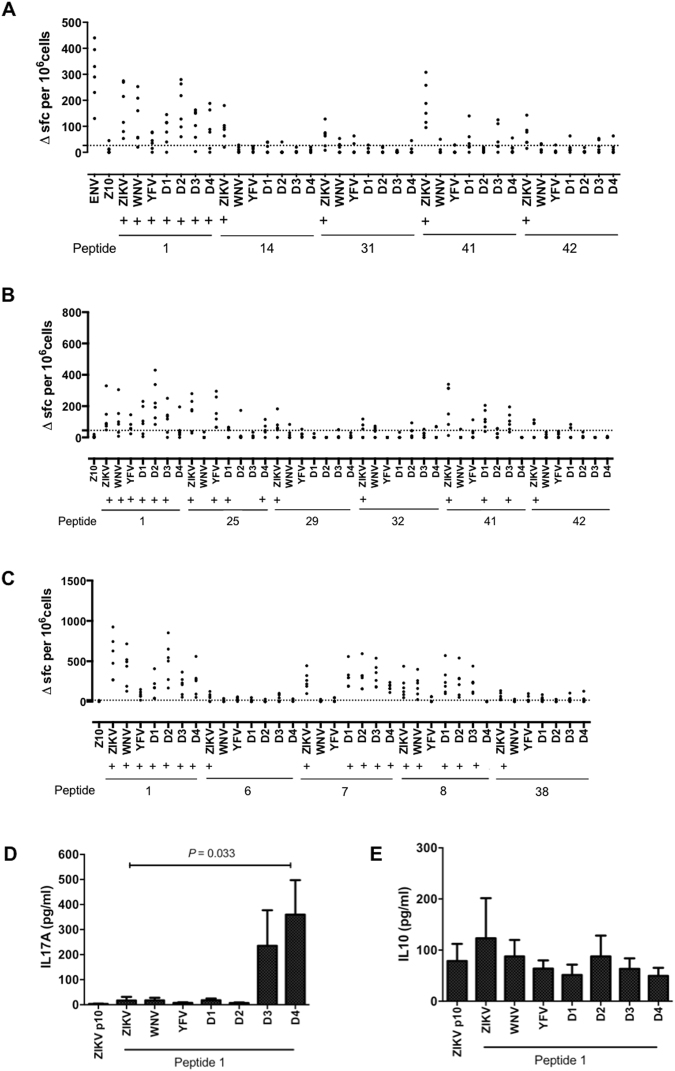
T cells responding to ZIKV envelope protein peptide 1 are crossreactive with variant peptides from other flavivirus species. Mice transgenic for (**A**) HLA-DR1 (DRB1*0101) n = 6, (**B**) HLA-DR15:01 (DRB1*1501) n = 6 and (**C**) HLA-DQ8 (DQB1*0301) n = 6 were primed with 25 μg of recombinant ZIKV envelope protein (Env). 10 days after immunisation, DLNs were assayed by IFNγ ELISpot for recall responses to the Env protein, Env peptide 10, Env peptides as shown and the corresponding peptide variants of west nile virus (WNV), yellow fever virus (YFV), dengue virus 1 (D1), dengue virus 2 (D2), dengue virus 3 (D3) and dengue virus 4 (D4). Data are plotted as number of spot forming cells (SFC) per 10^6^ cells for individual mice. Responses were considered positive (+) if the response was greater than 2 SD above the mean of the response in the absence of any antigen (shown as horizontal dotted line). HLA-DR1 ELISpot supernatants were collected prior to assay development and levels of (**D**) IL-17A and (**E**) IL-10 measured by ELISA. Data shown represent mean ± SEM. Statistical significance was determined using an unpaired t-test.

We screened for potential virus cross-reactive recognition by immunization with recombinant ZIKV Env, then screening T cell responses by IFNγ ELISpot for recognition of the selected ZIKV epitopes or the homologous sequences from WNV, YFV or DENV1–4. This was done using mice that were transgenic for HLA-DR1, HLA-DR1501 or HLA-DQ8 (Fig. 2A,B,C).

HLA-DR1 transgenic mice were immunized with ZIKV Env and popliteal lymph node cells analyzed *ex vivo* at d10 by IFNγ ELISpot for response to the immunodominant epitopes p1, p14, p31, p41 and p42, or to the variant versions we had identified in the sequences of WNV, YFV and DENV1–4 (Fig. 2A). With respect to p1, we observed significant responses to the cross-reactive epitopes from WNV, YFV and DENV1–4. In the case of YFV, the response was low (though positive by our criteria), showing the lowest sequence identity with the ZIKV p1 epitope, with matches at 12 of 20 residues. Tetramer analysis confirmed the presence of HLA-DRB1*0101 ZIKV p1 specific T cells and a high degree of cross reactivity with HLA-DRB1*0101 DENV4 p1 at the clonal level (Fig. S3). We also analyzed other cytokines produced during this response, to look for any indication that the cross-reactive peptides might act as an altered peptide ligand, with a modulated cytokine response relative to the ZIKV epitope. We found that the DENV4 p1 epitope (and to a lesser extent, the DENV3 p1 epitope) triggered an IL-17A response that was not present in the response to ZIKV p1 (Fig. 2D). Thus, stimulation of ZIKV Env primed T cells by the homologous DENV4 sequence shifts the response to a Th17 component. From the tools at www.iedb.org, the most likely minimal binding register is FVEGMSGGT (anchors underlined); considering the difference between the DENV4 p1 epitope and either the ZIKV or DENV1–3 epitopes, the variant residue that might influence T cell receptor (TCR) recognition (thereby mediating altered peptide ligand (APL) activity) is replacement of the methionine residue (a TCR contact), with the much smaller valine.

We defined no cross-reactive epitope recognition for the ZIKV Env p14, p31, or p42 epitopes. There was no significant cross-reactive epitope recognition for the ZIKV Env p41. When the same experiment was done immunizing HLA-DR1501 transgenics with ZIKV Env, we observed cross-reactive responses to p1 from WNV, YFV, and DENV1, 2 and 3 (Fig. 2B). There was also cross-reactive epitope recognition for the ZIKV Env immunodominant epitopes p25 and p41, but not for peptides p29, p32, and p42. With respect to p25, we observed significant responses to the cross-reactive epitopes from YFV and DENV4 and for p41 we observed significant responses to the cross-reactive epitopes from DENV1 and DENV3. Similar to HLA-DR1 transgenics, when Env immunized HLA-DQ8 transgenics were analyzed for ELISpot responses to the variant p1 peptides, significant responses were observed to each of the p1 variants, that is, from WNV, YFV, and DENV1–4 (Fig. 2C). There was also cross-reactive epitope recognition for the ZIKV Env immunodominant epitopes p7 (DENV1–4) and p8 (WNV and DENV1–3), but not for peptides p6 and p38. Thus, T cell activation by the cross-reactive variant peptides from other Flaviviruses may be to some extent dependent on the presenting HLA class II allele, and may be expected to differ between individuals.

In summary, these studies show examples where HLA class II transgenic T cells, primed to respond to ZIKV Env, can cross-reactively respond to the partially homologous sequences from other Flaviviruses, though results vary depending on the presenting HLA class II heterodimer. This was seen most extensively for ZIKV Env p1. In the example of p1 presentation by HLA-DR1, the DENV4 epitope functioned as an APL, eliciting an IL-17A response not seen with the ZIKV epitope. Tetramer analysis confirmed a high degree of cross reactivity at the clonal level.

### Antigen recognition, immunodominant epitopes and cross-reactivity in the CD4 T cell response to acute ZIKV infection

**Figure 3:**
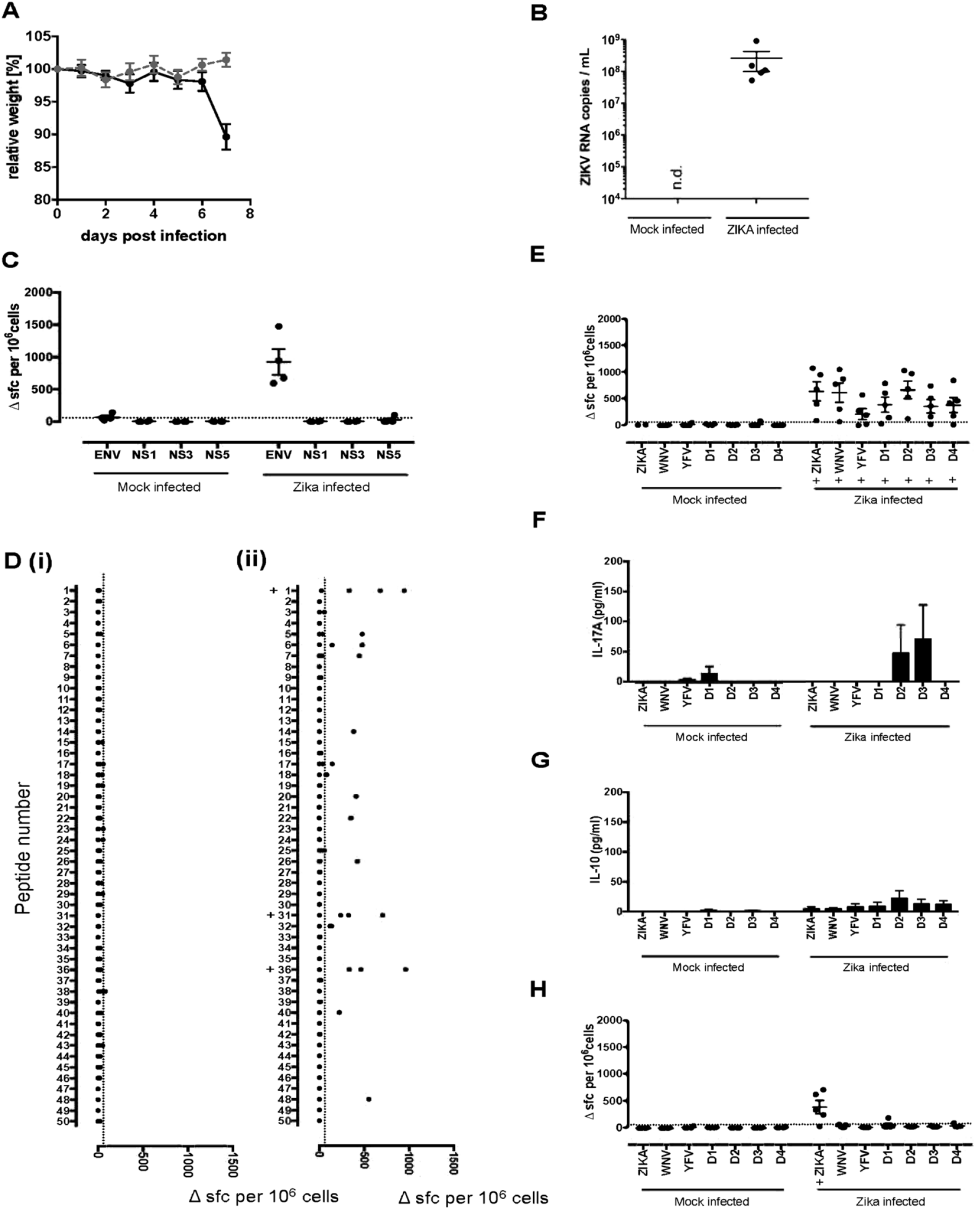
ZIKV virus infection in AG129 mice is associated with a strong T-cell response to ZIKV Env peptide 1 which is crossreactive with variant peptides from other flavivirus species. AG129 mice were (i) mock infected (n = 4) or (ii) infected intraperitoneally with 10^5^ FFU of ZIKV (PF13/251013-18) (n = 5). (**A**) Mock (grey circles) and ZIKV infected (black circles) mice were monitored daily for signs of weight loss and culled at 7 days post infection. (**B**) ZIKV RNA load was quantified by real time PCR. Splenocytes from both groups of mice were assayed by IFNγ ELISpot for recall responses to (**C**) ZIKV proteins Env, NS1, NS3 and NS5, (**D**) an overlapping panel of 50 Env peptides and flavivirus variant peptides from west nile virus (WNV), yellow fever virus (YFV), dengue virus 1 (D1), dengue virus 2 (D2), dengue virus 3 (D3) and dengue virus 4 (D4) for peptides 1 (**E**) and 31 (**H**). Data are plotted as number of spot forming cells (SFC) per 10^6^ cells for individual mice. Responses were considered positive (+) if the response was greater than 2 SD above the mean of the response in the absence of any antigen (shown as horizontal dotted line). ELISpot supernatants from peptide 1 variants were also collected prior to assay development and levels of IL-17A (**F**) and IL-10 (**G**) measured by ELISA. Data shown represent mean ± SEM.

We next investigated ZIKV antigen and epitope recognition in the context of acute ZIKV infection of adult AG129 mice. Mice were infected intraperitoneally with 10^5^ focus forming unit (FFU) Zika virus (PF13/251013-18) and were culled at day 7, having reached their clinical endpoint, including rapid weight loss and clinical signs of disease. Infected mice exhibited splenomegaly as well as marked liver abnormalities. ZIKV infection was confirmed by ZIKV RNA quantification (Fig. 3A,B).

Splenic T cells were screened by IFNγ ELISpot for their response to whole ZIKV Env, NS1, NS3 and NS5 proteins. A strong response was observed to Env but not to any of the non-structural proteins (Fig. 3C).

We then mapped the epitope specificity of AG129 splenic cells from d7 ZIKV infected mice. Strong Env peptide specific responses were seen against p1, p31 and p36 (Fig. 3D). We next determined whether priming in the context of acute infection stimulated a T cell response that recognized the cross-reactive p1 epitopes from WNV, YFV, and DENV1–4 (Fig. 3E). Strong responses were observed to each of these epitopes, the response to the WNV and DENV2 epitopes being of similar magnitude to the ZIKV p1 response. In this context, the response to DENV3 p1 (and to a lesser extent, DENV2 p1) yielded an APL IL-17A response not seen in response to ZIKV p1 (Fig. 3F) although this result did not achieve statistical significance. No APL IL-10 response to ZIKV p1 variant peptides was seen (Fig. 3G). No cross-reactive responses were identified to ZIKV Env p31 variants (Fig. 3H).

## Discussion

While the incidence of new Zika cases in Central and South America during the 2017 rainy season has, thus far, been lighter than 2016, Zika infection and the associated impacts with respect to congenital Zika syndrome continue to be a source of public health concern in more than 80 countries. Zika virus disease is notifiable in the USA, with more than 5000 cases notified, mainly of travelers returning from affected areas, with some hundreds resulting from local mosquito-borne transmission^19^. A very large number of candidate Zika vaccines, protective in animal models and based on diverse vaccination platforms, currently await trial^4^. While a number have already been demonstrated to confer protection from acute infection in non-human primate (NHP) challenge models, roll-out of Flavivirus vaccination programs can be non-trivial. Among the many issues is the fact that in many of the affected countries, ZIKV is one of several circulating Flaviviruses^9^. In some cases there is antigen cross-reactivity between the viruses. Whether such cross-reactivity, either ensuing from natural infection or from vaccination, might confer cross-protection and/or immune potentiation of enhanced disease is uncharted. Furthermore, the immunocompetent animal challenge models do not, in most cases, encompass the range of clinical phenotypes seen in human infection, so that protection from these remains hard to evaluate. Knowledge of protective adaptive immune mechanisms is, thus far, based on protection from acute experimental challenge, whereas understanding the consequences of exposure will require understanding of the control of viral persistence in diverse tissues^18^. At least as much as for any other virus genus, outcome after infection by Flaviviruses is driven be a complex interplay between viral pathogenicity and the multiple, protective or pathogenic components of host immunity. In any case, studies of adaptive immunity to ZIKV are in their infancy and there is a lack of any clear consensus of the correlates of protection.

Basic features of ZIKV protective immunity can be inferred from protective transfer studies in mice and from NHP vaccine studies. The data have recently been reviewed by Barouch and colleagues^4^. From the mouse studies, acute infection causes systemic activation of Natural killer (NK), CD4 and CD8 cells as well as the expected antibody response^15^. The ZIKV neutralizing antibody response has been documented and encompasses specificities that are cross-reactive with other Flaviviruses. Passive transfer of antibody can confer protection from acute challenge in the absence of a CD4 or CD8 response^4^. In support of the functional role of the CD8 response, CD8 ablated mice show enhanced susceptibility, while transfer of primed CD8 cells lowers viral load^14^. The T cell cytokine profile appears IFNγ rather than IL-17A focused, and an immunodominant epitope in Env has been described. A number of studies have described initial ZIKV T cells epitopes by screening responses of infected humans or mice to candidate epitopes predicted by the predictive tools in www.iedb.org. These have included CD4 and CD8 epitopes in Cap, Env, NS1, 2, 3, and 5^12–14^.

In the present study we started by immunizing mice that were endogenous class II-null but HLA transgenic for HLA-DR1 and DR4 with the ZIKV antigens Env, NS1, 3, or 5. Mapping the T cell epitopes within these antigens, we found a hierarchy of epitope density across the transgenic lines of Env > NS5 > NS3 > NS1. This is in contrast to epitope density in YFV vaccinees, where C > Env > NS2A = NS3 > NS1^20^, or DENV infection, where C > NS3 > NS5/NS2A > Env^21^. Overall, we have here been able to identify forty two ZIKV T cell epitopes. However, we note the caveat that CD4 epitopes predicted by HLA class II transgenic studies are not always found identically in humans of the same HLA type. At the initiation of this program, we had based our expectations to some extent on what was already known about human CD4 T cell epitopes from WNV, which showed precisely the same hierarchy^6^. The analogy with WNV T cell immunity also serves to remind that the anti-viral T cell response may encompass both protective and pathogenic phenotypes. In the WNV example, patients with neuroinvasive disease have raised numbers of specific T cells. Furthermore, these WNV responses included a CXCR3 + CCR4 + CCR6- T cell population, secreting IFNγ and IL-4, highly correlated with neuroinvasive disease^6^. On the other hand, neuroinflammation in JEV infection may depend on a pathogenic Th17 response under poor Treg control^7^. In terms of antibody responses to Flavivirus epitopes, anti-DENV antibodies can confer potentiation of virus uptake and disease exacerbation – ‘antibody dependent enhancement’ (ADE) – with respect either to subsequent ZIKV or DENV infection^8^.

Several of the defined epitopes were in regions of sequence showing relatively high sequence conservation with other Flaviviruses, including WNV, DENV1–4 and YFV. We therefore took the opportunity to investigate cross-reactive recognition by ZIKV-primed T cells of the variant sequences from other viruses. This was done for the variant Env sequences from WNV, YFV and DENV1–4 for p1, p14, p31, p41 and p42 first identified in HLA-DR1 transgenics. Cross-reactive responses were observed with each of these specificities, most commonly with the WNV sequence. Peptide p1 (AA 1–20) from ZIKV Env was noteworthy since it has now been defined to contain an immunodominant epitope that can be presented both through class I and class II for CD4 and CD8 responses (as we found in our Env primed mice). This Env 1–20 peptide (or core epitopes within this peptide) have been previously described in a number of other T cell studies, including humans infected with DENV1^22^, DENV3 immunized HLA-DR3 transgenic mice^23^ as well as CD4 responses of DENV2 Env-immunized mice^24^. The variant Env p1–20 sequences in WNV, YFV and DENV1–4 (as well as other closely related viruses such as Spondweni virus) each retain the residues xxCxGxxxRDFxEGxxGxxW. It is well established in other settings of T cell immunity, notably autoimmune or antiviral responses, that variant stimulatory peptides may interact differentially with TCR contact residues, thus acting as altered peptide ligands (APL) to deviate the phenotype of the effector response^25–28^. This was indeed the case for DENV variants of p1, showing an ability not seen with the wild-type ZIKV sequence, to elicit an IL-17A response. This APL effect was observed both after priming with Env protein and after acute ZIKV infection. Indeed, this peptide encompasses an epitope that has previously been described as a DENV1 T cell epitope that is cross-reactively recognized with DENV2^22^. It remains as yet unclear whether Th17 immunity may be neuropathogenic in ZIKV infection, as for JEV^7^. It might be predicted that there would be an increased risk of immunopathological effects if DENV infection (or vaccination) follows ZIKV infection. Th17 responses have been implicated in the immunopathogenesis of Dengue hemorrhagic fever^29^. Clearly, DENV1–4 and ZIKV show a highly overlapping distribution in Central and South America. Furthermore, there is potential overlap between ZIKV and WNV exposures in North America and in Asia. Possible impacts of T cell cross-reactivity between ZIKV and YFV merits some mention; it is unclear whether any such interaction could underpin the fact that the peak of ZIKV cases in Brazil during 2016 has been followed in 2017 by the sharpest peak for decades of severe YFV cases^30^.

Our findings serve to underline the point that many of the countries that have been facing a healthcare crisis of ZIKV infections are also at risk from several other, related Flaviviruses. This not only poses challenges to implementation of specific serodiagnsostics due to antigenic cross-reactivity, it also poses currently unanswered questions about the wider implications of cross-reactive immunity. At the most simplistic level, some of the cross-reactive overlaps may serve to confer a degree of cross-protection between infections. It could be speculated that such effects may underpin the observation that in Brazil, which has very high DENV seroprevalence, the vast majority of ZIKV exposures during the past seasons have been asymptomatic. However, the examples of ADE, both within DENV exposures and, potentially, between DENV and ZIKV, along with the possibility that cross-reactive stimulation may deviate T cell phenotypes to a more pathogenic profile, prompts a need for greater efforts to characterize these relationships, as well as greater vigilance and precision in our vaccination programs and understanding of their true correlates of protection.

## Materials and Methods

### Ethics statement

Mouse experiments were performed within U.K Home Office legislation under the terms of Home Office Project Licenses granted for this work under the “Animals (Scientific Procdures) Act 1986”. Local ethical review and formal approval was also obtained through the Imperial College Ethical Review Process Committee.

### Recombinant proteins and peptides

Sequences of the envelope protein (Env) and non-structural proteins NS1, NS3 and NS5 of the ZIKV were taken from a recent Brazilian isolate (GenBank accession no. AMH87239.1). Following codon optimisation, recombinant proteins were expressed in *Escherichia coli* and purified by His-Tag. The hydrophobic transmembrane domain (456–504) of Env was not included to improve protein expression, solubility and stability. (Biomatik, Cambridge, ON, Canada) (Supplementary Table 1). Synthetic peptides of 20 aa in length and overlapping by 10 aa were generated for each of the recombinant proteins (GL Biochem, Shanghai, China) (Supplementary Table 2). Flavivirus variants of Env peptides 1, 6, 7, 8, 14, 29, 31, 32, 38, 41 and 42 for WNV (accession no. AFJ05105.1), YFV (accession no. AIZ07887.1), DENV1 (accession no. AKQ00039.1), DENV2 (accession no. AKQ00040.1), DENV3 (accession no. ACO06174.1) and DENV4 (accession no. AKQ00037.1) were also synthesised (Table 4).

### HLA-peptide binding assays

HLA-DR heterdimers were purified from B cell lines by affinity purification on L243. Peptide binding was evaluated with competitive ELISA using an automated workstation. HLA heterodimers were incubated with biotinylated indicator peptide and serial dilutions of competitor peptide. As reference peptides, unlabelled forms of the biotinylated indicator peptide were used to assess validity in each experiment. The following reference sequences were used as labelled indicator peptides, and their IC_50_ values are indicated in parentheses: HA 306–318 (PKYVKQNTLKLAT) for DRB1*01:01 (3 nM), DRB1*04:01 (45 nM), DRB1*07:01 (28 nM) DRB1*1101 (21 nM) and DRB5*01:01 (10 nM), MT2-16 (AAKTIAYDEEARRGLE) for DRB1*03:01 (50 nM) and A3 152–166 (EAEQLRAYLDGTGVE) for DRB1*15:01 (40 nM). After 24–72 h incubation (37 °C), samples were neutralised with 50 μl 450 mM Tris-HCl (pH 7.5), 0.3% BSA, 1 mM DM buffer and applied to 96-well MaxiSorp ELISA plates (Nunc) coated with 10 μg/ml L243. Bound biotinylated peptide was detected by successive addition of streptavidin-alkaline phosphatase conjugate (GE Healthcare, Saclay, France) and 4-methylumbelliferyl phosphate substrate (Sigma, France). Emitted fluorescence was measured at 450 nm upon excitation at 365 nm. The peptide concentration preventing 50% binding of labelled peptide (IC50) was evaluated and data expressed as relative affinity: the ratio of the IC50 of test peptide to the IC50 of reference peptide. Mean ± SEM was calculated from two to three independent experiments. Relative affinities of 10 or less were considered high binders, and relative affinities of 10–100 were moderate binders.

### HLA transgenic mouse studies

This study used HLA class II transgenic mouse lines for the alleles HLA-DR1 (DRB1*0101), HLA-DR4 (DRB1*0401), HLA-DR1501 (DRB1*1501) and HLA-DQ8 (DQB1*0302), which were all maintained in the context of a homozygous knockout for murine H2-Aβ, as described previously^31–36^. Mice were maintained in individually ventilated cages and used in experiments as young adults. For T cell epitope mapping studies, mice were primed sub-cutaneously in one hind footpad with 25 μg recombinant protein emulsified in Hunters Titermax Gold adjuvant (Sigma-Aldrich). Ten days after immunisation the draining popliteal lymph nodes were removed and disaggregated into a single-cell suspension for ELISpot assays. The frequency of cells producing IFNγ in response to antigen was quantified by ELISpot using a murine IFN gamma ELISpot set kit (862.031.020, Diaclone; 2B Scientific, Oxon, U.K)^31–36^. Briefly, ELISpot plates (MSIPN4550, Merck Millipore, Massachusetts, USA) were coated with anti-mouse IFNγ capture antibody overnight before blocking with skimmed milk. Two x 10^5^ cells, from either lymph node or spleen, plus 25 μg/ml protein or peptide were added to wells in HL-1 serum free medium (Lonza, Slough, U.K), supplemented with L-glutamine and penicillin-streptomycin (ThermoFisher Scientific, U.K). Plates were incubated for 72 h, 37 °C with 5% CO2. Following assay development, spots were counted on an automated ELISpot reader (Autoimmun Diagnostika, Strasbourg, France). Response frequencies were expressed as Δ spot forming cells (SFC) per 10^6^ cells, with an epitope confirmed when the majority of immunised mice responded with a magnitude greater than the mean SFCs in the absence of any antigen +2 SD. Mean + 2 SD background SFC for each ELISpot is indicated by a dotted line. For some experiments, supernatants from ELISpot plates were collected prior to assay development and levels of IL-17A and IL-10 were quantified by ELISA using paired antibodies (DY421, R&D systems, Abingdon, UK) and (3432-1 H, Mabtech, Nacka Strand, Sweden) for IL-17A and IL-10 respectively.

### Flow Cytometry

Draining lymph node cells from HLA-DQ8 (DQB1*0302) mice primed 11 days previously with 25 μg/ml ZIKV Env protein were stimulated *in vitro* with 25 μg/ml of the relevant peptide, in the presence of 3 μg/ml Brefeldin A (Affymetrix eBioscience, USA) or with Brefeldin A alone. Cells were stimulated for 7 hours in RPMI medium supplemented with _L_-glutamine and Penicillin- streptomycin at 37 °C with 5% CO_2_. Stimulated cells were stained with the cell surface antibodies PE-anti mouse CD4 (clone GK1.5 from BD Pharmingen, USA), PE-Cy5 anti-mouse CD8a (clone 53–6.7 from Affymetrix eBioscience, USA), FITC anti-mouse CD3e (clone 145-2C11 from BD Pharmingen, USA) in PBS containing 10% fetal calf serum before fixation, permeabilisation and intracellular antibody staining with Alexa Fluor® 700 anti-mouse IFNγγ (clone XMG1.2 from BD Pharmingen, USA). R-phycoerythrin (R-PE) and Allophycocyanin (APC) labelled HLA-DRB1*0101 tetramers loaded with either the ZIKV Env p1 or the DENV4 Env p1 variant respectively were constructed as previously described^6,37^. Labelled tetramers containing no peptide were generated for use as controls^38^. 1 × 10^6^ DLN cells from ZIKV ENV primed HLA-DR1 transgenic mice were stained with 10 μg/ml of tetramer in 100 μl DMEM-10 media for 2.75 hours at 37 °C. Cells were then incubated with Fc block (BD Pharmingen, USA) for 15 minutes on ice followed by addition of Fluorescein conjugated anti-mouse CD4 (clone:GKL5, catalogue number:100406, Biolegend, USA) for a further 15 minutes. Data was collected using an Attune NxT flow cytometer (Thermofisher, UK) and analysed using FlowJo software (FlowJo, LLC, USA).

### Zika virus infection in AG129

AG129 mice were obtained from Marshall BioResources (Hull, UK). Animals were supplied with food and water *ad libitum* and monitored daily for signs of illness. Zika virus (PF13/251013-18, provided by European Commission Seventh Framework Program [FP7/2007-2013] for the DENFREE project under Grant Agreement n°282 378) was grown in C6/36 cells, and viral titers were determined by focus formation assay. Mice were infected intraperitoneally with 10^5^ FFU in a volume of 100 μL. They were weighed and clinically scored daily to monitor disease severity. After 7 d, mice were euthanized and blood and spleen was collected for further analysis.

### Zika virus RNA quantification

Zika virus RNA was quantified as previously described^39^. Briefly, RNA was isolated from plasma using a viral RNA extraction kit (Qiagen, UK) according to the manufacturer’s instructions and RNA was quantified using a Viia 7 real-time PCR system (Thermo Fisher Scientific, UK) using the TaqMan RNA-to-Ct 1-step kit (Thermo Fisher Scientific, UK). Plasmid pMA-T containing the complete 5’UTR of Zika virus was used for T7 polymerase *in vitro* transcription using the T7 Ribomax Express Large Scale RNA Production System (Promega, UK) and used as RNA standard.

## Electronic supplementary material

Supplementary Information

## References

[1] Lessler J (2016). Assessing the global threat from Zika virus. Science.

[2] Petersen LR, Jamieson DJ, Powers AM, Honein MA (2016). Zika Virus. N. Engl. J. Med..

[3] Wikan N, Smith DR (2016). Zika virus: history of a newly emerging arbovirus. Lancet Infect. Dis..

[4] Barouch DH, Thomas SJ, Michael NL (2017). Prospects for a Zika Virus Vaccine. Immunity.

[5] Lanteri MC (2009). Tregs control the development of symptomatic West Nile virus infection in humans and mice. J. Clin. Invest..

[6] James EA (2016). Neuroinvasive West Nile Infection Elicits Elevated and Atypically Polarized T Cell Responses That Promote a Pathogenic Outcome. PLoS Pathog..

[7] Kim JH (2016). CCR5 ameliorates Japanese encephalitis via dictating the equilibrium of regulatory CD4(+)Foxp3(+) T and IL-17(+)CD4(+) Th17 cells. J. Neuroinflammation.

[8] Screaton G, Mongkolsapaya J, Yacoub S, Roberts C (2015). New insights into the immunopathology and control of dengue virus infection. Nat. Rev. Immunol..

[9] Wilder-Smith A (2017). Epidemic arboviral diseases: priorities for research and public health. Lancet Infect. Dis..

[10] Panning M (2017). Zika virus serology: more diagnostic tests, more reliable answers?. EBioMedicine.

[11] Dejnirattisai W (2016). Dengue virus sero-cross-reactivity drives antibody-dependent enhancement of infection with Zika virus. Nat. Immunol..

[12] Stettler K (2016). Specificity, cross-reactivity, and function of antibodies elicited by Zika virus infection. Science.

[13] Pardy RD (2017). Analysis of the T Cell Response to Zika Virus and Identification of a Novel CD8+ T Cell Epitope in Immunocompetent Mice. PLoS Pathog..

[14] Elong Ngono A (2017). Mapping and Role of the CD8+ T Cell Response During Primary Zika Virus Infection in Mice. Cell Host Microbe.

[15] Manangeeswaran. M, Ireland DD, Verthelyi D (2016). Zika (PRVABC59) Infection Is Associated with T cell Infiltration and Neurodegeneration in CNS of Immunocompetent Neonatal C57Bl/6 Mice. PLoS Pathog.

[16] Wen J (2017). Identification of Zika virus epitopes reveals immunodominant and protective roles for dengue virus cross-reactive CD8+ T cells. Nat. Microbiol..

[17] Rivino L, Lim MQ (2017). CD4+ and CD8+ T-cell immunity to Dengue - lessons for the study of Zika virus. Immunology.

[18] Hirsch AJ (2017). Zika Virus infection of rhesus macaques leads to viral persistence in multiple tissues. PLoS Pathog..

[19] Centers for Disease Control and Prevention. Zika Cases in the United States. at https://www.cdc.gov/zika/geo/united-states.html (2017).

[20] James EA (2013). Yellow fever vaccination elicits broad functional CD4+ T cell responses that recognize structural and nonstructural proteins. J. Virol..

[21] Weiskopf D (2016). HLA-DRB1 Alleles Are Associated With Different Magnitudes of Dengue Virus-Specific CD4+ T-Cell Responses. J. Infect. Dis..

[22] Vaughan K, Greenbaum J, Blythe M, Peters B, Sette A (2010). Meta-analysis of all immune epitope data in the Flavivirus genus: inventory of current immune epitope data status in the context of virus immunity and immunopathology. Viral Immunol..

[23] Nascimento EJ (2013). Identification of conserved and HLA promiscuous DENV3 T-cell epitopes. PLoS Negl. Trop. Dis..

[24] Roehrig JT (1994). T-helper cell epitopes on the E-glycoprotein of dengue 2 Jamaica virus. Virology.

[25] Nicholson LB, Greer JM, Sobel RA, Lees MB, Kuchroo VK (1995). An altered peptide ligand mediates immune deviation and prevents autoimmune encephalomyelitis. Immunity.

[26] Kersh GJ, Allen PM (1996). Structural basis for T cell recognition of altered peptide ligands: a single T cell receptor can productively recognize a large continuum of related ligands. J. Exp. Med..

[27] Hunziker L (2002). Antagonistic variant virus prevents wild-type virus-induced lethal immunopathology. J. Exp. Med..

[28] Shorter SK (2016). Viral Escape Mutant Epitope Maintains TCR Affinity for Antigen yet Curtails CD8 T Cell Responses. PLoS One.

[29] Pagliari C (2016). Human kidney damage in fatal dengue hemorrhagic fever results of glomeruli injury mainly induced by IL17. J. Clin. Virol..

[30] Dyer O (2017). Yellow fever stalks Brazil in Zika’s wake. BMJ..

[31] Reynolds C (2015). T Cell Immunity to the Alkyl Hydroperoxide Reductase of Burkholderia pseudomallei: A Correlate of Disease Outcome in Acute Melioidosis. J. Immunol..

[32] Quigley KJ (2015). Chronic Infection by Mucoid Pseudomonas aeruginosa Associated with Dysregulation in T-Cell Immunity to Outer Membrane Porin F. Am. J. Respir. Crit. Care Med..

[33] Musson JA (2014). CD4+ T cell epitopes of FliC conserved between strains of Burkholderia: implications for vaccines against melioidosis and cepacia complex in cystic fibrosis. J. Immunol..

[34] Reynolds CJ (2014). The serodominant secreted effector protein of Salmonella, SseB, is a strong CD4 antigen containing an immunodominant epitope presented by diverse HLA class II alleles. Immunology.

[35] Ascough S (2014). Anthrax lethal factor as an immune target in humans and transgenic mice and the impact of HLA polymorphism on CD4+ T cell immunity. PLoS Pathog..

[36] Till SJ (2014). Peptide-induced immune regulation by a promiscuous and immunodominant CD4T-cell epitope of Timothy grass pollen: a role of Cbl-b and Itch in regulation. Thorax.

[37] Novak EJ, Liu AW, Nepom GT, Kwok WW (1999). MHC class II tetramers identify peptide-specific human CD4(+) T cells proliferating in response to influenza A antigen. J Clin Invest.

[38] Mattapallil, M. J. *et al*. Uveitis-associated epitopes of retinal antigens are pathogenic in the humanized mouse model of uveitis and identify autoaggressive T cells. **187**, 1977–85 (2011). 10.4049/jimmunol.1101247 PMC3150271 21765017

[39] Chan JF (2017). Improved detection of Zika virus RNA in human and animal specimens by a novel, highly sensitive and specific real-time RT-PCR assay targeting the 5′-untranslated region of Zika virus. Trop. Med. Int. Health..

