# Supplementary material for: T cell immunity to Zika virus targets immunodominant epitopes that show cross-reactivity with other Flaviviruses
Source: Sci Rep. 2018 Jan 12;8:672. doi: 10.1038/s41598-017-18781-1 (PMC5766511; doi:10.1038/s41598-017-18781-1)
Supplement: Supplementary file 1 — Supplementary Information [file 41598_2017_18781_MOESM1_ESM.pdf]

Supplementary Information

T cell immunity to Zika virus targets immunodominant epitopes that show cross-reactivity with other Flaviviruses

Reynolds CJ[1], Suleyman OM[1], Ortega-Prieto AM[1], Skelton JK[1], Bonnesoeur P[2], Blohm A[1], Carregaro V[3], Silva JS[3], James EA[4], Maillère B[2], Dorner M[1], Boyton RJ[1¶*], and Altmann DM[1¶*]

[1]Department of Medicine, Imperial College London, London, United Kingdom

[2]CEA-Saclay, Institute Frédéric Joliot, Gif Sur Yvette, France.

[3]Department of Biochemistry and Immunology, University of São Paulo Ribeirão Preto, Brazil.

[4]Benaroya Research Institute at Virginia Mason, Seattle, Washington, United States of America.

¶ co-senior authors

Supplementary Table 1. ZIKV protein sequences: Env, NS1, NS3, and NS5

ZIKV protein	AA sequence
Env	IRCIGVSNRD FVEGMSGGTW VDVVLEHGGC VTVMAQDKPT<br>VDIELVTTTV SNMAEVRSYC YEASISDMAS DSRCPTQGEA<br>YLDKQSDTQY VCKRTLVDRG WGNGCGLFGK GSLVTCAKFA<br>CSKKMTGKSI QPENLEYRIM LSVHGSQHSG MIVNDTGHET<br>DENRAKVEIT PNSPRAEATL GGFGSLGLDC EPRTGLDFSD<br>LYYLTMNNKH WLVHKEWFHD IPLPWHAGAD TGTPHWNNKE<br>ALVEFKDAHA KRQTVVVLGS QEGAVHTALA GALEAEMDGA<br>KGRLSSGHLK CRLKMDKLRL KGVSYSLCTA AFTFTKIPAE<br>TLHGTVTVEV QYAGTDGPCK VPAQMAVDMQ TLTPVGRLIT<br>ANPVITESTE NSKMMLELDP PFGDSYIVIG VGEKKITHHW<br>HRSGSTIGKA FEATVRGAKR MAVLGDTAWD FGSVGGALNS<br>LGKGIHQIFG AAFKSLFGGM SWFSQILIGT LLMWLGLNTK<br>NGSISLMCLA LGGVLIFLST AVSA
NS1	DVGCSVDFSK KETRCGTGVF VYNDVEAWRD RYKYHPDSPR<br>RLAAAVKQAW EDGICGISSV SRMENIMWRS VEGELNAILE<br>ENGVQLTVVV GSVKNPMWRG PQRLPVPVNE LPHGWKAWGK<br>SYFVRAAKTN NSFVVDGDTL KECPLKHRAW NSFLVEDHGF<br>GVFHTSVWLK VREDYSLECD PAVIGTAVKG KEAVHSDLGY<br>WIESEKNDTW RLKRAHLIEM KTCEWPKSHT LWTDGIEESD<br>LIIPKSLAGP LSHHNTREGY RTQMKGPWHS EELEIRFEEC<br>PGTKVHVEET CGTRGPSLRS TTASGRVIEE WCCRECTMPP<br>LSFRAKDGCW YGMEIRPRKE PESNLVRSMV TA
NS3	SGALWDVPAP KEVKKGETTD GVYRVMTRRL LGSTQVGVGV<br>MQEGVFHTMW HVTKGSALRS GEGRLDPYWG DVKQDLVSYC<br>GPWKLDAAWD GHSEVQLLAV PPGERARNIQ TLPGIFKTKD<br>GDIGAVALDY PAGTSGSPIL DKCGRVIGLY GNGVVIKNGS<br>YVSAITQGRR EEETPVECFE PSMLKKKQLT VLDLHPGAGK<br>TRRVLPEIVR EAIKTRLRTV ILAPTRVVAA EMEEALRGLP<br>VRYMTTAVNV THSGTEIVDL MCHATFTSRL LQPIRVPNYN<br>LYIMDEAHFT DPSSIAARGY ISTRVEMGEA AAIFMTATPP<br>GTRDAFPDSN SPIMDTEVEV PERAWSSGFD WVTDHSGKTV<br>WFVPSVRNGN EIAACLTKAG KRVIQLSRKT FETEFQKTKH<br>QEWDFVVTTD ISEMGANFKA DRVIDSRRCL KPVILDGERV<br>ILAGPMPVTH ASAAQRRGRI GRNPNKPGDE YLYGGGCAET<br>DEDHAHWLEA RMLLDNIYLQ DGLIASLYRP EADKVAAIEG<br>EFKLRTEQRK TFVELMKRGD LPVWLAYQVA SAGITYTDRR<br>WCFDGTTNNT IMEDSVPAEV WTRHGEKRVL KPRWMDARVC

SDHAALKSFK EFAAGKR

NS5	GGGTGETLGE KWKARLNQMS ALEFYSYKKS GITEVCREEA<br>RRALKDGVAT GGHAVSRGSA KLRWLVERGY LQPYGKVIDL<br>GCGRGGWSYY AATIRKVQEV KGYTKGGPGH EEPVLVQSYG<br>WNIVRLKSGV DVFHMAAEPC DTLLCDIGES SSSPEVEEAR<br>TLRVLSMVGD WLEKRPGAFC IKVLCPYTST MMETLERLQR<br>RYGGGLVRVP LSRNSTHEMY WVSGAKSNTI KSVSTTSQLL<br>LGRMDGPRRP VKYEEDVNLG SGTRAVVSCA EAPNMKIIGN<br>RIERIRSEHA ETWFFDENHP YRTWAYHGSY EAPTQGSASS<br>LINGVVRLLS KPWDVVTGVT GIAMTDTTPY GQQRVFKEKV<br>DTRVPDPQEG TRQVMSMVSS WLWKELGKHK RPRVCTKEEF<br>INKVRSNAAL GAIFEEEKEW KTAVEAVNDP RFWALVDKER<br>EHHLRGECQS CVYNMMGKRE KKQGEFGKAK GSRAIWYMWL<br>GARFLEFEAL GFLNEDHWMG RENSGGGVEG LGLQRLGYVL<br>EEMSRIPGGR MYADDTAGWD TRISRFDLEN EALITNQMEK<br>GHRALALAII KYTYQNKVVK VLRPAEKGKT VMDIISRQDQ<br>RGSGQVVTYA LNTFTNLVVQ LIRNMEAEEV LEMQDLWLLR<br>RSEKVTNWLQ SNGWDRLKRM AVSGDDCVVK PIDDRFAHAL<br>RFLNDMGKVR KDTQEWKPST GWDNWEEVPF CSHHFNKLHL<br>KDGRSIVVPC RHQDELIGRA RVSPGAGWSI RETACLAKSY<br>AQMWQLLYFH RRDLRLMANA ICSSVPVDWV PTGRTTWSIH<br>GKGEWMTTED MLVVWNRVWI EENDHMEDKT PVTKWTDIPY<br>LGKREDLWCG SLIGHRPRTT WAENIKNTVN MVRRIIGDEE<br>KYMDYLSTQV RYLGEEGSTP GVL

Supplementary Table 2. ZIKV protein antigen peptide panels for NS1, NS3, NS5, and Env

ZIKV NS1 (10AA OVERLAP)

PEPTIDE NO.	peptide name	peptide sequence
1	Zika NS1 [1-20]	DVGCSVDFSKKETRCGTGVF
2	Zika NS1 [11-30]	KETRCGTGVFVYNDVEAWRD
3	Zika NS1 [21-40]	VYNDVEAWRDRYKYHPDSPR
4	Zika NS1 [31-50]	RYKYHPDSPRRLAAAVKQAW
5	Zika NS1 [41-60]	RLAAAVKQAWEDGICGISSV
6	Zika NS1 [51-70]	EDGICGISSVSRMENIMWRS
7	Zika NS1 [61-80]	SRMENIMWRSVEGELNAILE
8	Zika NS1 [71-90]	VEGELNAILEENGVQLTVVV
9	Zika NS1 [81-100]	ENGVQLTVVVGSVKNPMWRG
10	Zika NS1 [91-110]	GSVKNPMWRGPQRLPVPVNE
11	Zika NS1 [101-120]	PQRLPVPVNELPHGWKAWGK
12	Zika NS1 [111-130]	LPHGWKAWGKSYFVRAAKTN
13	Zika NS1 [121-140]	SYFVRAAKTNNSFVVDGDTL
14	Zika NS1 [131-150]	NSFVVDGDTLKECPLKHRAW
15	Zika NS1 [141-160]	KECPLKHRAWNSFLVEDHGF
16	Zika NS1 [151-170]	NSFLVEDHGFGVFHTSVWLK
17	Zika NS1 [161-180]	GVFHTSVWLKVREDYSLECD
18	Zika NS1 [171-190]	VREDYSLECDPAVIGTAVKG
19	Zika NS1 [181-200]	PAVIGTAVKGKEAVHSDLGY
20	Zika NS1 [191-210]	KEAVHSDLGYWIESEKNDTW
21	Zika NS1 [201-220]	WIESEKNDTWRLKRAHLIEM
22	Zika NS1 [211-230]	RLKRAHLIEMKTCEWPKSHT
23	Zika NS1 [221-240]	KTCEWPKSHTLWTDGIEESD
24	Zika NS1 [231-250]	LWTDGIEESDLIIPKSLAGP
25	Zika NS1 [241-260]	LIIPKSLAGPLSHHNTREGY
26	Zika NS1 [251-270]	LSHHNTREGYRTQMKGPWHS
27	Zika NS1 [261-280]	RTQMKGPWHSEELEIRFEEC
28	Zika NS1 [271-290]	EELEIRFEECPGTKVHVEET
29	Zika NS1 [281-300]	PGTKVHVEETCGTRGPSLRS
30	Zika NS1 [291-310]	CGTRGPSLRSTTASGRVIEE
31	Zika NS1 [301-320]	TTASGRVIEEWCCRECTMPP
32	Zika NS1 [311-330]	WCCRECTMPPLSFRAKDGCW
33	Zika NS1 [321-340]	LSFRAKDGCWYGMEIRPRKE
34	Zika NS1 [331-350]	YGMEIRPRKEPESNLVRSMV
35	Zika NS1 [333-352]	MEIRPRKEPESNLVRSMVTA

ZIKV NS3 (10AA OVERLAP)

PEPTIDE NO.	peptide name	peptide sequence
1	Zika NS3 [1-20]	SGALWDVPAPKEVKKGETTD
2	Zika NS3 [11-30]	KEVKKGETTDGVYRVMTRRL
3	Zika NS3 [21-40]	GVYRVMTRRLLGSTQVGVGV
4	Zika NS3 [31-50]	LGSTQVGVGVMQEGVFHTMW
5	Zika NS3 [41-60]	MQEGVFHTMWHVTKGSALRS
6	Zika NS3 [51-70]	HVTKGSALRSGEGRLDPYWG
7	Zika NS3 [61-80]	GEGRLDPYWGDVKQDLVSYC
8	Zika NS3 [71-90]	DVKQDLVSYCGPWKLDAAWD
9	Zika NS3 [81-100]	GPWKLDAAWDGHSEVQLLAV
10	Zika NS3 [91-110]	GHSEVQLLAVPPGERARNIQ
11	Zika NS3 [101-120]	PPGERARNIQTLPGIFKTKD
12	Zika NS3 [111-130]	TLPGIFKTKDGDIGAVALDY
13	Zika NS3 [121-140]	GDIGAVALDYPAGTSGSPIL
14	Zika NS3 [131-150]	PAGTSGSPILDKCGRVIGLY
15	Zika NS3 [141-160]	DKCGRVIGLYGNGVVIKNGS
16	Zika NS3 [151-170]	GNGVVIKNGSYVSAITQGRR
17	Zika NS3 [161-180]	YVSAITQGRREEETPVECFE
18	Zika NS3 [171-190]	EEETPVECFEPSMLKKKQLT
19	Zika NS3 [181-200]	PSMLKKKQLTVLDLHPGAGK
20	Zika NS3 [191-210]	VLDLHPGAGKTRRVLPEIVR
21	Zika NS3 [201-220]	TRRVLPEIVREAIKTRLRTV
22	Zika NS3 [211-230]	EAIKTRLRTVILAPTRVVAA
23	Zika NS3 [221-240]	ILAPTRVVAAEMEEALRGLP
24	Zika NS3 [231-250]	EMEEALRGLPVRYMTTAVNV
25	Zika NS3 [241-260]	VRYMTTAVNVTHSGTEIVDL
26	Zika NS3 [251-270]	THSGTEIVDLMCHATFTSRL
27	Zika NS3 [261-280]	MCHATFTSRLLQPIRVPNYN
28	Zika NS3 [271-290]	LQPIRVPNYNLYIMDEAHFT
29	Zika NS3 [281-300]	LYIMDEAHFTDPSSIAARGY
30	Zika NS3 [291-310]	DPSSIAARGYISTRVEMGEA
31	Zika NS3 [301-320]	ISTRVEMGEAAAIFMTATPP
32	Zika NS3 [311-330]	AAIFMTATPPGTRDAFPDSN
33	Zika NS3 [321-340]	GTRDAFPDSNSPIMDTEVEV
34	Zika NS3 [331-350]	SPIMDTEVEVPERAWSSGFD
35	Zika NS3 [341-360]	PERAWSSGFDWVTDHSGKTV
36	Zika NS3 [351-370]	WVTDHSGKTVWFVPSVRNGN
37	Zika NS3 [361-380]	WFVPSVRNGNEIAACLTKAG
38	Zika NS3 [371-390]	EIAACLTKAGKRVIQLSRKT
39	Zika NS3 [381-400]	KRVIQLSRKTFETEFQKTKH
40	Zika NS3 [391-410]	FETEFQKTKHQEWDFVVTTD
41	Zika NS3 [401-420]	QEWDFVVTTDISEMGANFKA

**42**	Zika NS3 [411-430]	ISEMGANFKADRVIDSRRCL
**43**	Zika NS3 [421-440]	DRVIDSRRCLKPVILDGERV
**44**	Zika NS3 [431-450]	KPVILDGERVILAGPMPVTH
**45**	Zika NS3 [441-460]	ILAGPMPVTHASAAQRRGRI
**46**	Zika NS3 [451-470]	ASAAQRRGRIGRNPNKPGDE
**47**	Zika NS3 [461-480]	GRNPNKPGDEYLYGGGCAET
**48**	Zika NS3 [471-490]	YLYGGGCAETDEDHAHWLEA
**49**	Zika NS3 [481-500]	DEDHAHWLEARMLLDNIYLQ
**50**	Zika NS3 [491-510]	RMLLDNIYLQDGLIASLYRP
**51**	Zika NS3 [501-520]	DGLIASLYRPEADKVAAIEG
**52**	Zika NS3 [511-530]	EADKVAAIEGEFKLRTEQRK
**53**	Zika NS3 [521-540]	EFKLRTEQRKTFVELMKRGD
**54**	Zika NS3 [531-550]	TFVELMKRGDLPVWLAYQVA
**55**	Zika NS3 [541-560]	LPVWLAYQVASAGITYTDRR
**56**	Zika NS3 [551-570]	SAGITYTDRRWCFDGTTNNT
**57**	Zika NS3 [561-580]	WCFDGTTNNTIMEDSVPAEV
**58**	Zika NS3 [571-590]	IMEDSVPAEVWTRHGEKRVL
**59**	Zika NS3 [581-600]	WTRHGEKRVLKPRWMDARVC
**60**	Zika NS3 [591-610]	KPRWMDARVCSDHAALKSFK
**61**	Zika NS3 [598-617]	RVCSDHAALKSFKEFAAGKR

ZIKV NS5 (10AA OVERLAP)

PEPTIDE NO.	peptide name	peptide sequence
1	Zika NS5 [1-20]	GGGTGETLGEKWKARLNQMS
2	Zika NS5 [11-30]	KWKARLNQMSALEFYSYKKS
3	Zika NS5 [21-40]	ALEFYSYKKSGITEVCREEA
4	Zika NS5 [31-50]	GITEVCREEARRALKDGVAT
5	Zika NS5 [41-60]	RRALKDGVATGGHAVSRGSA
6	Zika NS5 [51-70]	GGHAVSRGSAKLRWLVERGY
7	Zika NS5 [61-80]	KLRWLVERGYLQPYGKVIDL
8	Zika NS5 [71-90]	LQPYGKVIDLGCGRGGWSYY
9	Zika NS5 [81-100]	GCGRGGWSYYAATIRKVQEV
10	Zika NS5 [91-110]	AATIRKVQEVKGYTKGGPGH
11	Zika NS5 [101-120]	KGYTKGGPGHEEPVLVQSYG
12	Zika NS5 [111-130]	EEPVLVQSYGWNIVRLKSGV
13	Zika NS5 [121-140]	WNIVRLKSGVDVFHMAAEPC
14	Zika NS5 [131-150]	DVFHMAAEPCDTLLCDIGES
15	Zika NS5 [141-160]	DTLLCDIGESSSSPEVEEAR
16	Zika NS5 [151-170]	SSSPEVEEARTLRVLSMVGD
17	Zika NS5 [161-180]	TLRVLSMVGDWLEKRPGAFC
18	Zika NS5 [171-190]	WLEKRPGAFCIKVLCPYTST
19	Zika NS5 [181-200]	IKVLCPYTSTMMETLERLQR
20	Zika NS5 [191-210]	MMETLERLQRRYGGGLVRVP
21	Zika NS5 [201-220]	RYGGGLVRVPLSRNSTHEMY
22	Zika NS5 [211-230]	LSRNSTHEMYWVSGAKSNTI
23	Zika NS5 [221-240]	WVSGAKSNTIKSVSTTSQLL
24	Zika NS5 [231-250]	KSVSTTSQLLLGRMDGPRRP
25	Zika NS5 [241-260]	LGRMDGPRRPVKYEEDVNLG
26	Zika NS5 [251-270]	VKYEEDVNLGSGTRAVVSCA
27	Zika NS5 [261-280]	SGTRAVVSCAEAPNMKIIGN
28	Zika NS5 [271-290]	EAPNMKIIGNRIERIRSEHA
29	Zika NS5 [281-300]	RIERIRSEHAETWFFDENHP
30	Zika NS5 [291-310]	ETWFFDENHPYRTWAYHGSY
31	Zika NS5 [301-320]	YRTWAYHGSYEAPTQGSASS
32	Zika NS5 [311-330]	EAPTQGSASSLINGVVRLLS
33	Zika NS5 [321-340]	LINGVVRLLSKPWDVVTGVT
34	Zika NS5 [331-350]	KPWDVVTGVTGIAMTDTTPY
35	Zika NS5 [341-360]	GIAMTDTTPYGQQRVFKEKV
36	Zika NS5 [351-370]	GQQRVFKEKVDTRVPDPQEG
37	Zika NS5 [361-380]	DTRVPDPQEGTRQVMSMVSS
38	Zika NS5 [371-390]	TRQVMSMVSSWLWKELGKHK
39	Zika NS5 [381-400]	WLWKELGKHKRPRVCTKEEF
40	Zika NS5 [391-410]	RPRVCTKEEFINKVRSNAAL
41	Zika NS5 [401-420]	INKVRSNAALGAIFEEEKEW

**42**	Zika NS5 [411-430]	GAIFEEEKEWKTAVEAVNDP
**43**	Zika NS5 [421-440]	KTAVEAVNDPRFWALVDKER
**44**	Zika NS5 [431-450]	RFWALVDKEREHHLRGECQS
**45**	Zika NS5 [441-460]	EHHLRGECQSCVYNMMGKRE
**46**	Zika NS5 [451-470]	CVYNMMGKREKKQGEFGKAK
**47**	Zika NS5 [461-480]	KKQGEFGKAKGSRAIWYMWL
**48**	Zika NS5 [471-490]	GSRAIWYMWLGARFLEFEAL
**49**	Zika NS5 [481-500]	GARFLEFEALGFLNEDHWMG
**50**	Zika NS5 [491-510]	GFLNEDHWMGRENSGGGVEG
**51**	Zika NS5 [501-520]	RENSGGGVEGLGLQRLGYVL
**52**	Zika NS5 [511-530]	LGLQRLGYVLEEMSRIPGGR
**53**	Zika NS5 [521-540]	EEMSRIPGGRMYADDTAGWD
**54**	Zika NS5 [531-550]	MYADDTAGWDTRISRFDLEN
**55**	Zika NS5 [541-560]	TRISRFDLENEALITNQMEK
**56**	Zika NS5 [551-570]	EALITNQMEKGHRALALAII
**57**	Zika NS5 [561-580]	GHRALALAIIKYTYQNKVVK
**58**	Zika NS5 [571-590]	KYTYQNKVVKVLRPAEKGKT
**59**	Zika NS5 [581-600]	VLRPAEKGKTVMDIISRQDQ
**60**	Zika NS5 [591-610]	VMDIISRQDQRGSGQVVTYA
**61**	Zika NS5 [601-620]	RGSGQVVTYALNTFTNLVVQ
**62**	Zika NS5 [611-630]	LNTFTNLVVQLIRNMEAEEV
**63**	Zika NS5 [621-640]	LIRNMEAEEVLEMQDLWLLR
**64**	Zika NS5 [631-650]	LEMQDLWLLRRSEKVTNWLQ
**65**	Zika NS5 [641-660]	RSEKVTNWLQSNGWDRLKRM
**66**	Zika NS5 [651-670]	SNGWDRLKRMAVSGDDCVVK
**67**	Zika NS5 [661-680]	AVSGDDCVVKPIDDRFAHAL
**68**	Zika NS5 [671-690]	PIDDRFAHALRFLNDMGKVR
**69**	Zika NS5 [681-700]	RFLNDMGKVRKDTQEWKPST
**70**	Zika NS5 [691-710]	KDTQEWKPSTGWDNWEEVPF
**71**	Zika NS5 [701-720]	GWDNWEEVPFCSHHFNKLHL
**72**	Zika NS5 [711-730]	CSHHFNKLHLKDGRSIVVPC
**73**	Zika NS5 [721-740]	KDGRSIVVPCRHQDELIGRA
**74**	Zika NS5 [731-750]	RHQDELIGRARVSPGAGWSI
**75**	Zika NS5 [741-760]	RVSPGAGWSIRETACLAKSY
**76**	Zika NS5 [751-770]	RETACLAKSYAQMWQLLYFH
**77**	Zika NS5 [761-780]	AQMWQLLYFHRRDLRLMANA
**78**	Zika NS5 [771-790]	RRDLRLMANAICSSVPVDWV
**79**	Zika NS5 [781-800]	ICSSVPVDWVPTGRTTWSIH
**80**	Zika NS5 [791-810]	PTGRTTWSIHGKGEWMTTED
**81**	Zika NS5 [801-820]	GKGEWMTTEDMLVVWNRVWI
**82**	Zika NS5 [811-830]	MLVVWNRVWIEENDHMEDKT
**83**	Zika NS5 [821-840]	EENDHMEDKTPVTKWTDIPY
**84**	Zika NS5 [831-850]	PVTKWTDIPYLGKREDLWCG
**85**	Zika NS5 [841-860]	LGKREDLWCGSLIGHRPRTT

**86**	Zika NS5 [851-870]	SLIGHRPRTTWAENIKNTVN
**87**	Zika NS5 [861-880]	WAENIKNTVNMVRRIIGDEE
**88**	Zika NS5 [871-890]	MVRRIIGDEEKYMDYLSTQV
**89**	Zika NS5 [881-900]	KYMDYLSTQVRYLGEEGSTP
**90**	Zika NS5 [884-903]	DYLSTQVRYLGEEGSTPGVL

ZIKV ENVELOPE PROTEIN Env (10AA OVERLAP)

PEPTIDE NO.	peptide name	peptide sequence
1	Zika ENV [1-20]	IRCIGVSNRDFVEGMSGGTW
2	Zika ENV [11-30]	FVEGMSGGTWVDVVLEHGGC
3	Zika ENV [21-40]	VDVVLEHGGCVTVMAQDKPT
4	Zika ENV [31-50]	VTVMAQDKPTVDIELVTTTV
5	Zika ENV [41-60]	VDIELVTTTVSNMAEVRSYC
6	Zika ENV [51-70]	SNMAEVRSYCYEASISDMAS
7	Zika ENV [61-80]	YEASISDMASDSRCPTQGEA
8	Zika ENV [71-90]	DSRCPTQGEAYLDKQSDTQY
9	Zika ENV [81-100]	YLDKQSDTQYVCKRTLVDRG
10	Zika ENV [91-110]	VCKRTLVDRGWGNGCGLFGK
11	Zika ENV [101-120]	WGNGCGLFGKGSLVTCAKFA
12	Zika ENV [111-130]	GSLVTCAKFACSKKMTGKSI
13	Zika ENV [121-140]	CSKKMTGKSIQPENLEYRIM
14	Zika ENV [131-150]	QPENLEYRIMLSVHGSQHSG
15	Zika ENV [141-160]	LSVHGSQHSGMIVNDTGHET
16	Zika ENV [151-170]	MIVNDTGHETDENRAKVEIT
17	Zika ENV [161-180]	DENRAKVEITPNSPRAEATL
18	Zika ENV [171-190]	PNSPRAEATLGGFGSLGLDC
19	Zika ENV [181-200]	GGFGSLGLDCEPRTGLDFSD
20	Zika ENV [191-210]	EPRTGLDFSDLYYLTMNNKH
21	Zika ENV [201-220]	LYYLTMNNKHWLVHKEWFHD
22	Zika ENV [211-230]	WLVHKEWFHDIPLPWHAGAD
23	Zika ENV [221-240]	IPLPWHAGADTGTPHWNNKE
24	Zika ENV [231-250]	TGTPHWNNKEALVEFKDAHA
25	Zika ENV [241-260]	ALVEFKDAHAKRQTVVVLGS
26	Zika ENV [251-270]	KRQTVVVLGSQEGAVHTALA
27	Zika ENV [261-280]	QEGAVHTALAGALEAEMDGA
28	Zika ENV [271-290]	GALEAEMDGAKGRLSSGHLK
29	Zika ENV [281-300]	KGRLSSGHLKCRLKMDKLRL
30	Zika ENV [291-310]	CRLKMDKLRLKGVSYSLCTA
31	Zika ENV [301-320]	KGVSYSLCTAAFTFTKIPAE
32	Zika ENV [311-330]	AFTFTKIPAETLHGTVTVEV
33	Zika ENV [321-340]	TLHGTVTVEVQYAGTDGPCK
34	Zika ENV [331-350]	QYAGTDGPCKVPAQMAVDMQ
35	Zika ENV [341-360]	VPAQMAVDMQTLTPVGRLIT
36	Zika ENV [351-370]	TLTPVGRLITANPVITESTE
37	Zika ENV [361-380]	ANPVITESTENSKMMLELDP
38	Zika ENV [371-390]	NSKMMLELDPPFGDSYIVIG
39	Zika ENV [381-400]	PFGDSYIVIGVGEKKITHHW
40	Zika ENV [391-410]	VGEKKITHHWHRSGSTIGKA
41	Zika ENV [401-420]	HRSGSTIGKAFEATVRGAKR

**42**	Zika ENV [411-430]	FEATVRGAKRMAVLGDTAWD
**43**	Zika ENV [421-440]	MAVLGDTAWDFGSVGGALNS
**44**	Zika ENV [431-450]	FGSVGGALNSLGKGIHQIFG
**45**	Zika ENV [441-460]	LGKGIHQIFGAAFKSLFGGM
**46**	Zika ENV [451-470]	AAFKSLFGGMSWFSQILIGT
**47**	Zika ENV [461-480]	SWFSQILIGTLLMWLGLNTK
**48**	Zika ENV [471-490]	LLMWLGLNTKNGSISLMCLA
**49**	Zika ENV [481-500]	NGSISLMCLALGGVLIFLST
**50**	Zika ENV [485-504]	SLMCLALGGVLIFLSTAVSA

S1 Fig. CD4 T cell epitopes to ZIKV non-structural proteins NS1, NS3 and NS5 in HLA-DR1 and HLA-DR4 transgenic mice.

Mice transgenic for HLA-DR1 (DRB1*0101) (i) and HLA-DR4 (DRB1*0401) (ii) were footpad primed with 25 μg of recombinant ZIKV proteins NS1 (A), NS3 (B) and NS5 (C). Ten days after immunisation, draining lymph node (DLN) cells were assayed by IFNγ ELISpot for recall responses to the immunising protein and to an overlapping panel of 20mer peptides spanning the length of each protein. Data shown are for n=6 mice per group except for (A ii) and (B i) which were n=4 and n=5 respectively. Data are plotted as number of spot forming cells (SFC) per 10^6 cells for individual mice. Responses were considered positive (+) if the response was greater than 2 SD above the mean of the response in the absence of any antigen (shown as horizontal dotted line).

HLA-DR1 (DRB1*0101)

A (i)

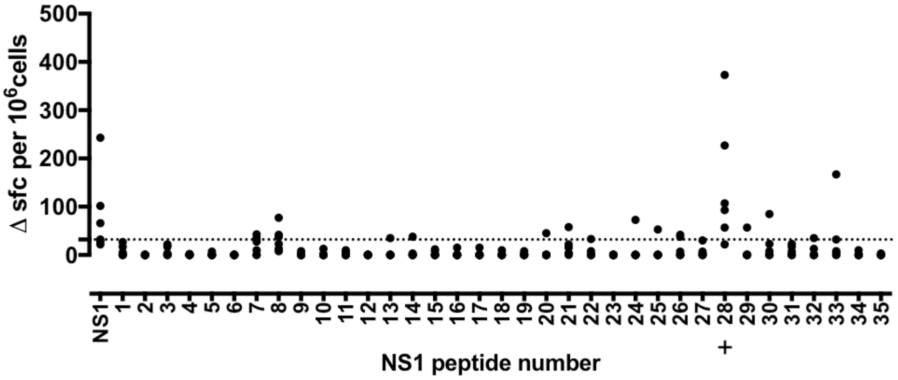

B (i)

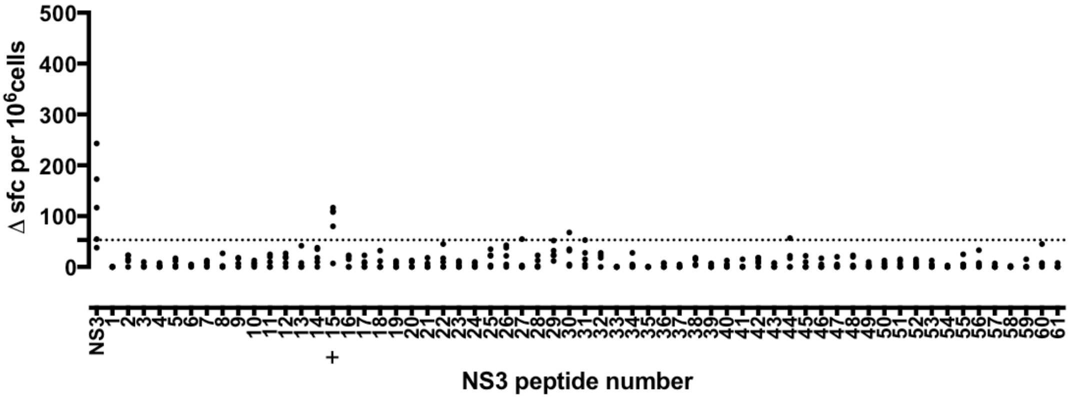

C (i)

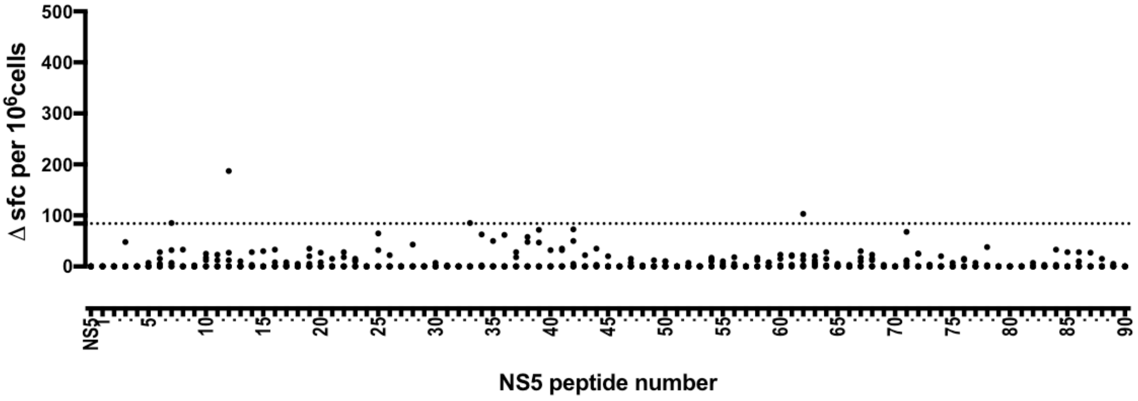

HLA-DR4 (DRB1*0401)

A (ii)

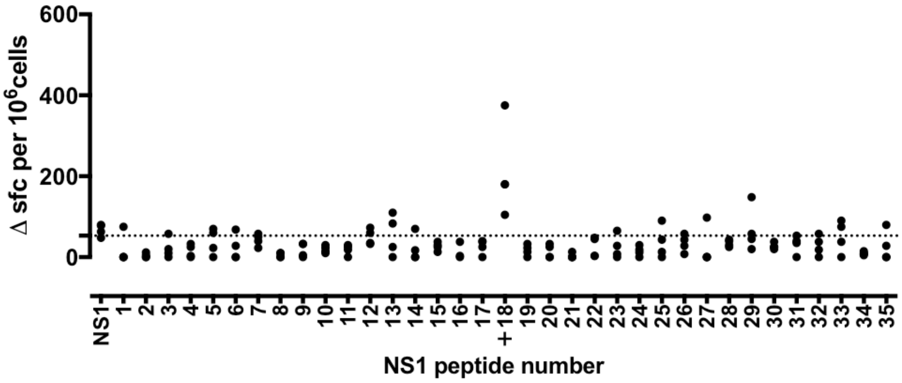

B (ii)

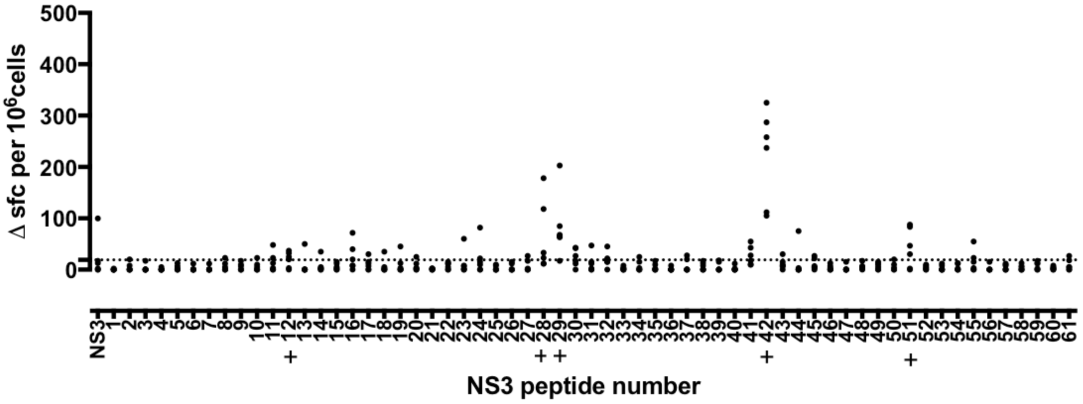

C (ii)

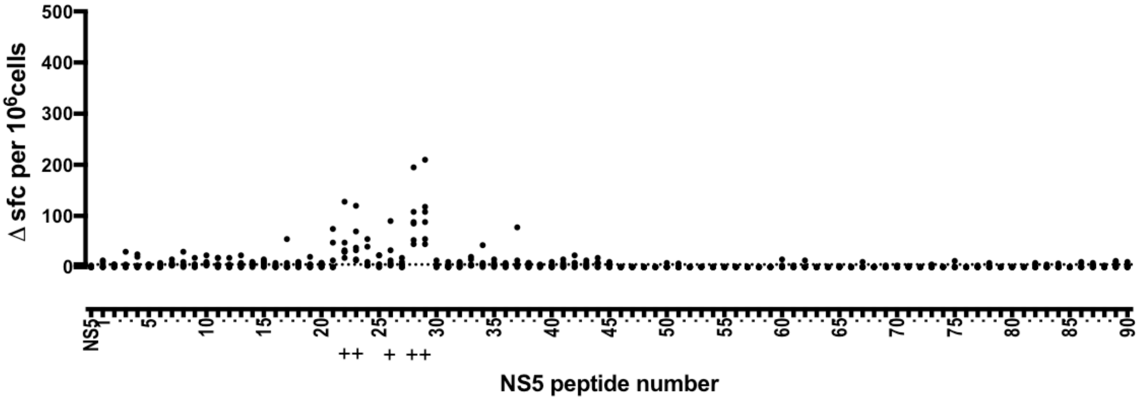

S2 Fig. CD4$^+$ and CD8$^+$ T cells respond to peptide 1 and variants of ZIKV Env

Mice transgenic for HLA-DQ8 (DQB1*0302) n=3 were primed with 25 μg of recombinant ZIKV envelope protein (Env). 11 days after immunisation, DLNs were cultured *in vitro* for 7 hours, in the presence of brefeldin A, with 25 μg/ml of Env peptide 10, Env peptide 1 or the peptide 1 variants of west nile virus (WNV), yellow fever virus (YFV), dengue virus 1 (D1), dengue virus 2 (D2), dengue virus 3 (D3) or dengue virus 4 (D4). Cells were stained for the cell surface markers CD3, CD4 and CD8 and intracellularly for the production of IFNγ. Data are plotted as the percentage of (A) CD4$^+$ T cells or (B) CD8$^+$ T cells producing IFNγ. Data shown represent mean ± SEM.

S2 Fig. CD4^{+} and CD8^{+} T cells respond to peptide 1 and variants of ZIKV Env

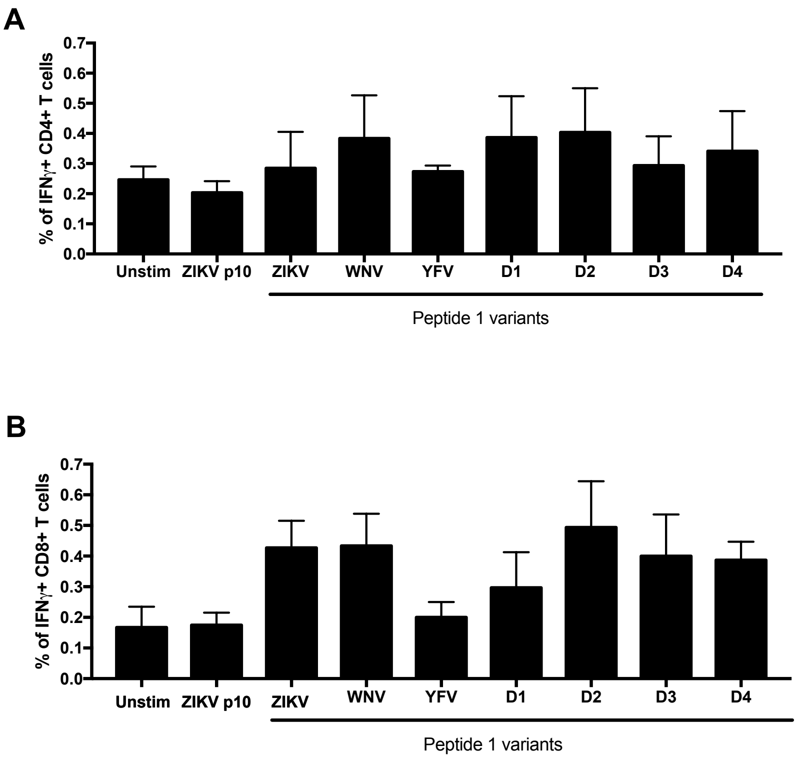

S3 Fig. Draining lymph node (DLN) cells from HLA-DR1 transgenic mice primed with ZIKV Env and stained with HLA class II tetramers loaded with peptide confirm the presence of ZIKV Env p1 specific T cells and demonstrate a high degree of cross-reactivity with the DENV4 p1 variant peptide at the clonal level.

HLA-DR1 (DRB1*0101) mice were primed sub-cutaneously in one hind footpad with 25μg of recombinant protein emulsified in Hunters Titermax Gold adjuvant (Sigma-Aldrich, UK). Fourteen days post immunisation, the draining popliteal lymph node was removed and disaggregated into a single-cell suspension. Cells were stained with HLA-DRB1*0101 tetramers loaded with either no peptide, ZIKV Env p1 or the DENV4 Env p1 variant followed by staining with an anti-mouse CD4 FITC antibody. (A) Samples were gated on live lymphocytes followed by $CD4^+$ cells before analysis of (B) ZIKV Env p1 specific T cells, (C) DENV4 Env p1 variant specific T cells and (D) dual ZIKV Env p1 and DENV4 Env p1 specific T cells.

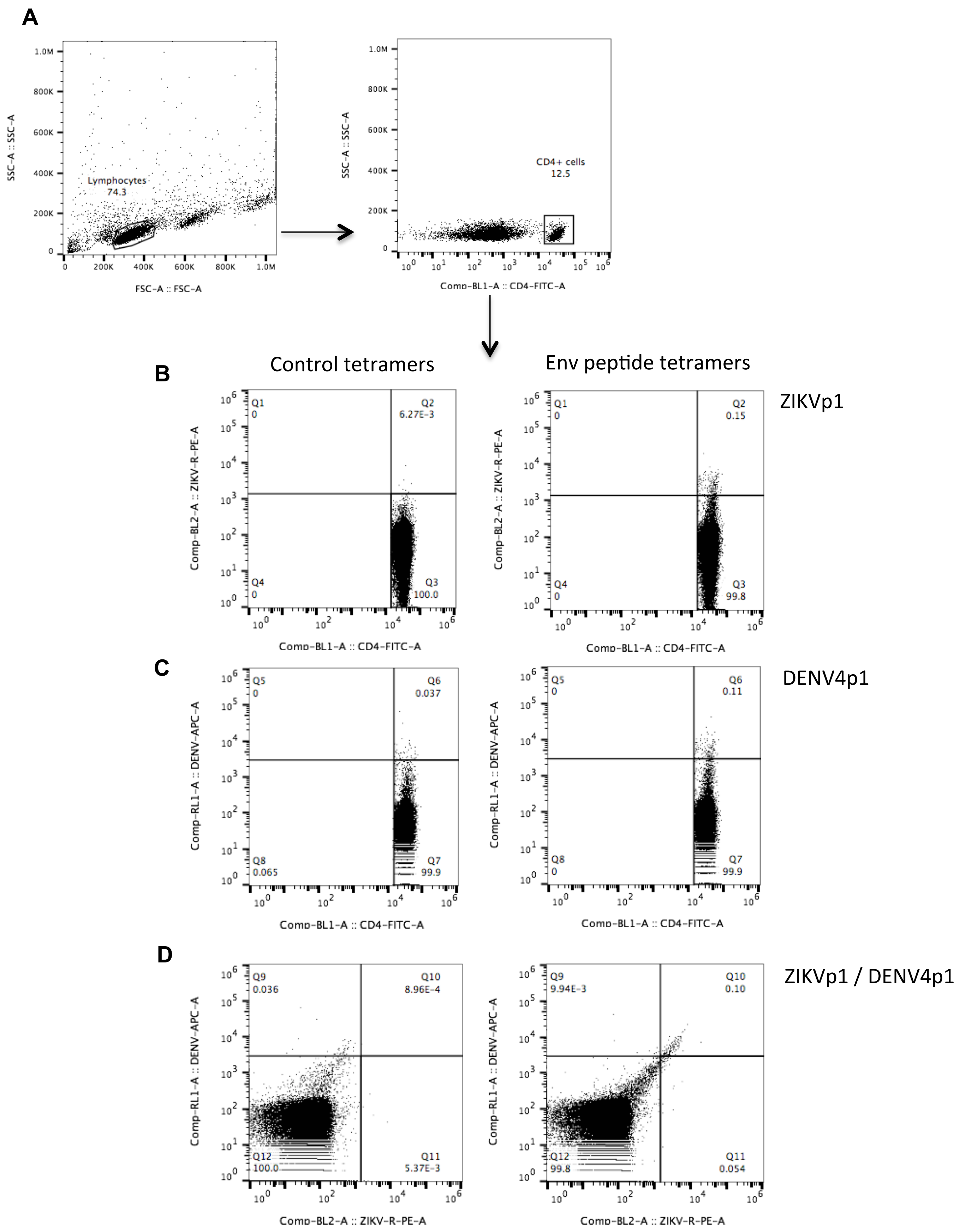
A
Lymphocytes
74.3
SSC-A :: SSC-A
FSC-A :: FSC-A
CD4+ cells
12.5
Comp-BL1-A :: CD4-FITC-A
B
Control tetramers
Env peptide tetramers
ZIKVp1
Comp-BL2-A :: ZIKV-R-PE-A
Q1 0
Q2 6.27E-3
Q4 0
Q3 100.0
Q1 0
Q2 0.15
Q4 0
Q3 99.8
C
DENV4p1
Comp-RL1-A :: DENV-APC-A
Q5 0
Q6 0.037
Q8 0.065
Q7 99.9
Q5 0
Q6 0.11
Q8 0
Q7 99.9
D
ZIKVp1 / DENV4p1
Q9 0.036
Q10 8.96E-4
Q12 100.0
Q11 5.37E-3
Q9 9.94E-3
Q10 0.10
Q12 99.8
Q11 0.054
Comp-BL2-A :: ZIKV-R-PE-A
